# First Access to Tetraazadiindenopyrenes via Selective Pyrrole Cyclization of Phenyl‐Substituted Tetraazapyrene Derivatives on Au(111)

**DOI:** 10.1002/smsc.70288

**Published:** 2026-05-08

**Authors:** Isabelle Kolly, Gema Navarro‐Marín, Robert Häner, Silvio Decurtins, Ernst Meyer, Ulrich Aschauer, Rémy Pawlak, Shi‐Xia Liu

**Affiliations:** ^1^ Department of Chemistry Biochemistry and Pharmaceutical Sciences W. Inäbnit Laboratory for Molecular Quantum Materials and WSS‐Research Center for Molecular Quantum Systems University of Bern Bern Switzerland; ^2^ Department of Physics WSS‐Research Center for Molecular Quantum Systems University of Basel Basel Switzerland; ^3^ Department of Chemistry and Physics of Materials University of Salzburg Salzburg Austria

**Keywords:** computational chemistry, cyclodehydrogenation, nitrogen heterocycles, on‐surface reactions, scanning probe microscopy

## Abstract

In stark contrast to cyclodehydrogenation (CDH) for constructing graphene nanoribbons through C─C or C─N bond formation, selective pyrrole cyclization via on‐surface chemistry remains exceptionally rare and largely unexplored. To the best of our knowledge, this study reports the first demonstration of the sequential CDH of phenyl‐substituted tetraazapyrene (TAP) derivatives on an Au(111) surface, providing the first access to tetraazadiindenopyrenes via selective pyrrole cyclization followed by C–C coupling. By varying the number and position of the phenyl substituents, we reveal how molecular geometry and aromaticity dictate the cyclization pathway, promoting C─N bond formation over the well‐established C–C coupling embedded within the TAP framework. Low‐temperature noncontact atomic force microscopy (nc‐AFM) and differential conductance spectroscopy, complemented by density functional theory (DFT) calculations, not only provide an unambiguous structural assignment of the final products but also offer mechanistic insight into the sequential C–N and C–C cyclization processes.

## Graphical abstract

Contrary to long‐held expectations of on‐surface chemistry, tetraphenyl‐substituted tetraazapyrene undergoes selective pyrrole cyclization and C–C coupling on Au(111), forming a distinctive N‐doped nanographene consisting of fused tetraazadiindenopyrene and phenanthrene cores with embedded pyridinic and graphitic nitrogen. Combined experimental and theoretical evidence supports the C–N coupling over the C–C coupling during cyclodehydrogenation.

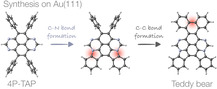

## 1 Introduction

Nitrogen heterocycles are ubiquitous in a wide range of pharmaceuticals, agrochemicals, natural products, and organic polymers due to their appealing structural features and versatile chemical properties [1, 2, 3]. Similarly, nitrogen‐rich heterocycles have attracted interest in the field of high‐energy‐density materials [4, 5]. These properties largely stem from the lone pair of electrons on the nitrogen atom, which either remains localized or participates in aromatic π‐conjugation, leading to distinct bonding configurations. Cyclic triimidazoles serve as a representative example, incorporating both types of N atoms within their molecular framework, namely pyridinic‐N, which is typically bound to two adjacent atoms and has a localized lone pair, and pyrrolic‐ or graphitic‐N, which is bound to three adjacent atoms and has its lone pair delocalized as part of the π‐conjugation [6, 7, 8, 9].

Given the rising demand for N‐heterocycles, C─N bond construction has become a central focus in modern organic synthesis [10, 11, 12, 13, 14]. Among the various synthetic methodologies, the hydroamination of alkenes stands out as a well‐established catalytic method for the C─N bond formation in solution [13, 14, 15]. However, extending this approach to surface‐confined nanostructures poses a major challenge in terms of both synthesis and elucidation of the mechanism, particularly when targeting N‐doping in graphene‐like nanomaterials. Nitrogen incorporation can significantly modulate the bandgap, enhance charge carrier mobility, and introduce new functionalities, leading to intrinsic optical, electrical, and magnetic properties. Therefore, considerable efforts have been devoted to characterize N‐doped nanographenes and graphene nanoribbons prepared by surface‐assisted Ullman‐type reaction and CDH using low‐temperature scanning tunneling microscopy (STM) and noncontact atomic force microscopy (nc‐AFM) [16, 17, 18, 19, 20, 21, 22, 23, 24, 25, 26, 27, 28, 29, 30, 31, 32, 33, 34, 35, 36]. With this bottom‐up approach, pyridinic‐N [32, 33, 34, 35, 36] has been observed more frequently than graphitic‐N in nanographenes [16, 28, 29, 30, 31]. This finding can be accounted for by the good synthetic accessibility of precursors that have been prefunctionalized with edge‐positioned pyridinic‐N groups, particularly through thermally driven surface‐assisted C–C coupling reactions. The formation of graphitic‐N is more demanding since it involves oxidative C−N coupling embedded within the aromatic polycyclic hydrocarbon (PAH). It results in atomic‐precise modulation of electronic properties induced by the participation of the lone pair of electrons on nitrogen in the π‐conjugation of the PAH [33].

In contrast to the Ullmann coupling on metal surfaces, the incorporation of graphitic‐N into PAHs was only achieved in 2020 with the successful C─N bond formation through surface‐assisted thermal CDH. In this pioneering study, tetraazateranthene was synthesized via CDH of dianthryl pyrazino[2,3‐*g*]quinoxalines on Au(111) [28]. Shortly thereafter, 11,11,12,12‐tetraphenyl‐1,4,5,8‐tetraazaanthraquinodimethane was found to undergo a distinct transformation of heterocyclic segregation on the same surface to furnish diquinolinodiazapentacene [37]. This transformation involved thermally induced cyclization via C─N bond formation and subsequent C─N bond cleavage within the pyrazine ring, accompanied by the elimination of C_2_H_4_. As a result, only pyridinic nitrogen atoms are incorporated into diquinolinodiazapentacene, in stark contrast to tetraazateranthene, which contains exclusively graphitic‐N atoms. These results underscore how subtle variations in molecular structure can determine different nitrogen configurations in the resulting N‐PAHs. It is also worth noting that, among C─N bond formation reactions, there are only two reported cases of selective pyrrole ring closure from nonpyrrolic precursors via surface‐assisted reactions [15, 38].

**FIGURE 1:**
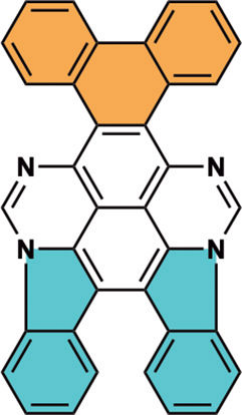
The chemical structure of a teddy‐bear‐shaped molecule **4** composed of fused tetraazadiindenopyrene (indene units in cyan) and phenanthrene (in orange) cores.

Our keen interest in 1,3,6,8‐tetraazapyrene (**TAP**) arises from its unique molecular architecture and intrinsic electronic properties [39, 40, 41]. In recent work, we have demonstrated that 4,5,9,10‐tetrabromo‐1,3,6,8‐tetraazapyrene (**TBTAP**) can form a stable radical on the superconducting Pb(111) surface [42, 43, 44], opening avenues for the emergence of a two‐dimensional spin lattice and switchable topological superconductivity [45]. In order to expand the scope of these studies and their potential applications, we are investigating the on‐surface chemical reaction of 4,5,9,10‐tetraphenyl‐1,3,6,8‐tetraazapyrene (**4P‐TAP**) on Au(111), resulting in the formation of a N‐PAH (**4**) with a characteristic teddy‐bear shape (Figure 1). This transformation proceeds via sequential C–N and C–C cyclization steps. To gain insight into the reaction mechanism, three diphenyl TAP isomers were synthesized by a one‐pot reaction involving the cascade bromination of the TAP core, followed by Suzuki coupling with phenylboronic acid. All these newly synthesized TAPs were fully characterized using NMR spectroscopy and high‐resolution mass spectrometry (HR‐MS). The structures of the resulting N‐PAHs on Au(111) were unambiguously identified by scanning probe microscopy and differential conductance spectroscopy, as well as corroborated by density functional theory (DFT) calculations.

## 2 Results and Discussion

### 2.1 Synthesis of Phenyl‐Substituted TAP Derivatives

**SCHEME 1:**
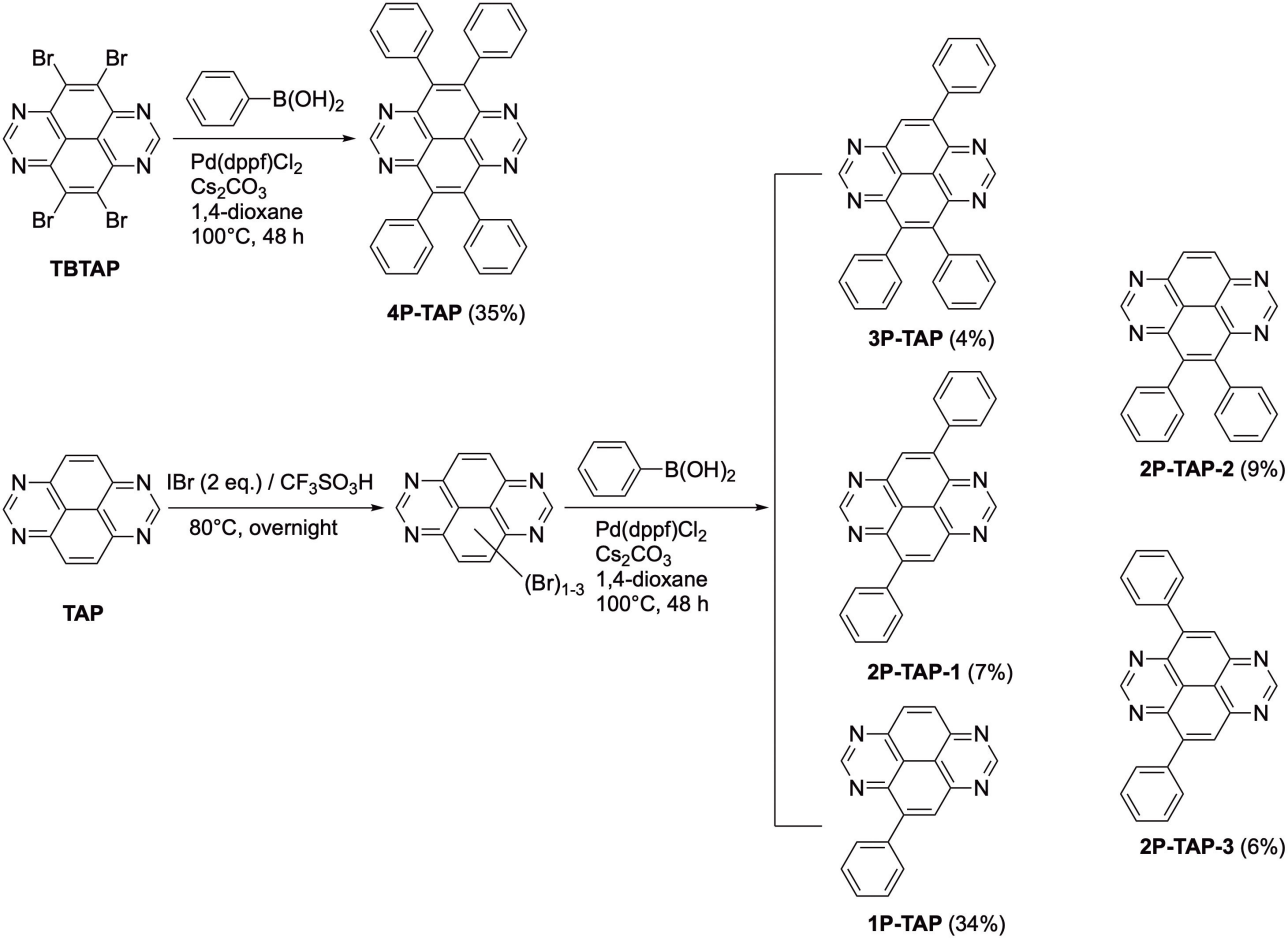
Synthesis of a series of phenyl‐substituted TAP derivatives.

Both **TAP** and **TBTAP** were synthesized according to established procedures reported in the literature [40, 41]. **4P‐TAP** was readily prepared in a yield of 35% via a Pd‐catalyzed Suzuki coupling reaction between **TBTAP** and phenylboronic acid. As depicted in Scheme 1, a facile and efficient synthetic protocol for cascade bromination of **TAP** was developed using iodine monobromide (IBr). Similar to the stepwise chlorination of 2,7‐di‐*tert*‐butyl‐1,3,6,8‐tetraazapyrene with ICl [46], treatment of **TAP** with 2 equivalents of IBr in trifluoromethanesulfonic acid at 80°C overnight afforded a mixture of mono‐, di‐, and tri‐brominated products. It was, however, impossible to separate them by careful column chromatography due to their strong tendency to aggregate. To overcome this challenge, the crude brominated mixture was subjected directly to Suzuki coupling with phenylboronic acid, which allowed us to isolate the corresponding mono‐, di‐, and tri‐phenyl TAP derivatives. Remarkably, three analytically pure di‐substituted TAP isomers, **2P‐TAP‐1**, **2P‐TAP‐2**, and **2P‐TAP‐3** were successfully obtained in a one‐pot reaction. These new compounds are characterized by NMR and HR‐MS, as shown in the Supplementary Section.

### 2.2 Experimental Identification of C─N Bond Formation in TAP Derivatives

To investigate the stepwise CDH of **2P‐TAP‐1,** **2P‐TAP‐2**, **2P‐TAP‐3**, and **4P‐TAP**, we performed a series of experiments in which each derivative was deposited under ultra‐high vacuum (UHV) conditions from a Knudsen cell onto an atomically clean Au(111) surface maintained at room temperature. The resulting large supramolecular assemblies were characterized by STM (Figure S1). A subsequent annealing of the substrate to 200°C induces intramolecular ring closures. Bond‐resolved STM/AFM images at *T* = 4.5 K using CO‐terminated tips (see Methods) were employed to visualize their chemical structures in detail.

**FIGURE 2:**
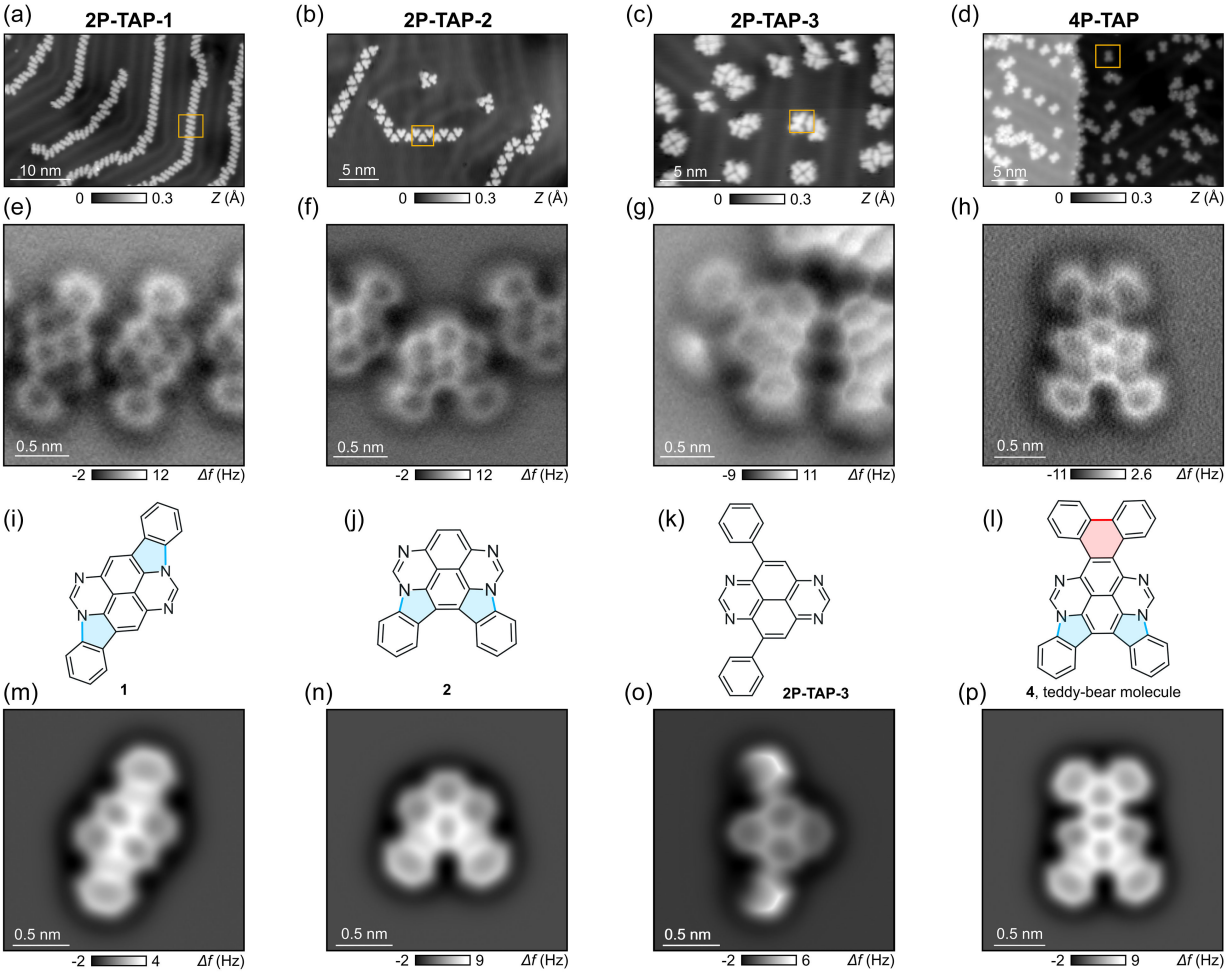
On‐surface reactions of phenyl‐substituted TAP derivatives on Au(111). (a–d) Large‐scale STM images after annealing **2P‐TAP‐1**, **2P‐TAP‐2**, **2P‐TAP‐3**, and **4P‐TAP** derivatives on Au(111) (voltage *V* = −0.1 V, tunneling current *I* = 1 pA). (e–h) Constant‐height AFM images of the reacted products (oscillation amplitude A_osc _= 50 pm). Images were acquired in the orange rectangles of (a–d), respectively. The cyclodehydrogenation of **2P‐TAP‐1**, **2P‐TAP‐2**, and **4P‐TAP** leads to products **1**, **2**, and **4**, respectively, after the formation of two C─N single bonds. **2P‐TAP‐3** does not react under the same experimental conditions. (i–l) Interpreted chemical structures of the reacted products **1**, **2**, and **4**, as well as unreacted **2P‐TAP‐3**, based on AFM images. Red and blue bonds represent newly formed C─C and C─N single bonds, forming two aromatic pyrrole units (blue) and a benzene ring (red). (m–p) Simulated AFM images using the probe‐particle model [47].

Figure 2a–d shows large‐scale STM images obtained after annealing each TAP derivative at 200°C. Long chain structures were observed for the reacted products of **2P‐TAP‐1** and **2P‐TAP‐2**, as well as for the unreacted **2P‐TAP‐3** molecules (Figure 2a–c), while the reacted product of **4P‐TAP** predominantly appears as isolated species or small aggregates (Figure 2d). In contrast to the molecules in the not yet annealed supramolecular assemblies (Figure S1), all resulting compounds, except for unreacted **2P‐TAP‐3**, are planarized by thermally induced cyclization on the surface. Corresponding constant‐height AFM images, acquired at the orange rectangles in Figure 2a–d, were used to confirm the presence of newly formed intramolecular C─N and C─C single bonds (Figure 2e–h).

The experimental AFM images of the reaction products can be interpreted using the molecular structures shown in Figure 2i–l. The newly formed C─C and C─N single bonds and resulting rings are colored in red and blue, respectively. The structure coordinates from DFT were also used to simulate AFM images with the probe‐particle model [47] (Figure 2m‐p), yielding a good agreement with their experimental counterparts and confirming the structural assignment. Under the same experimental conditions, the **2P‐TAP‐3** molecules show no signs of cyclization, as confirmed by the AFM image in Figure 2g.

**SCHEME 2:**
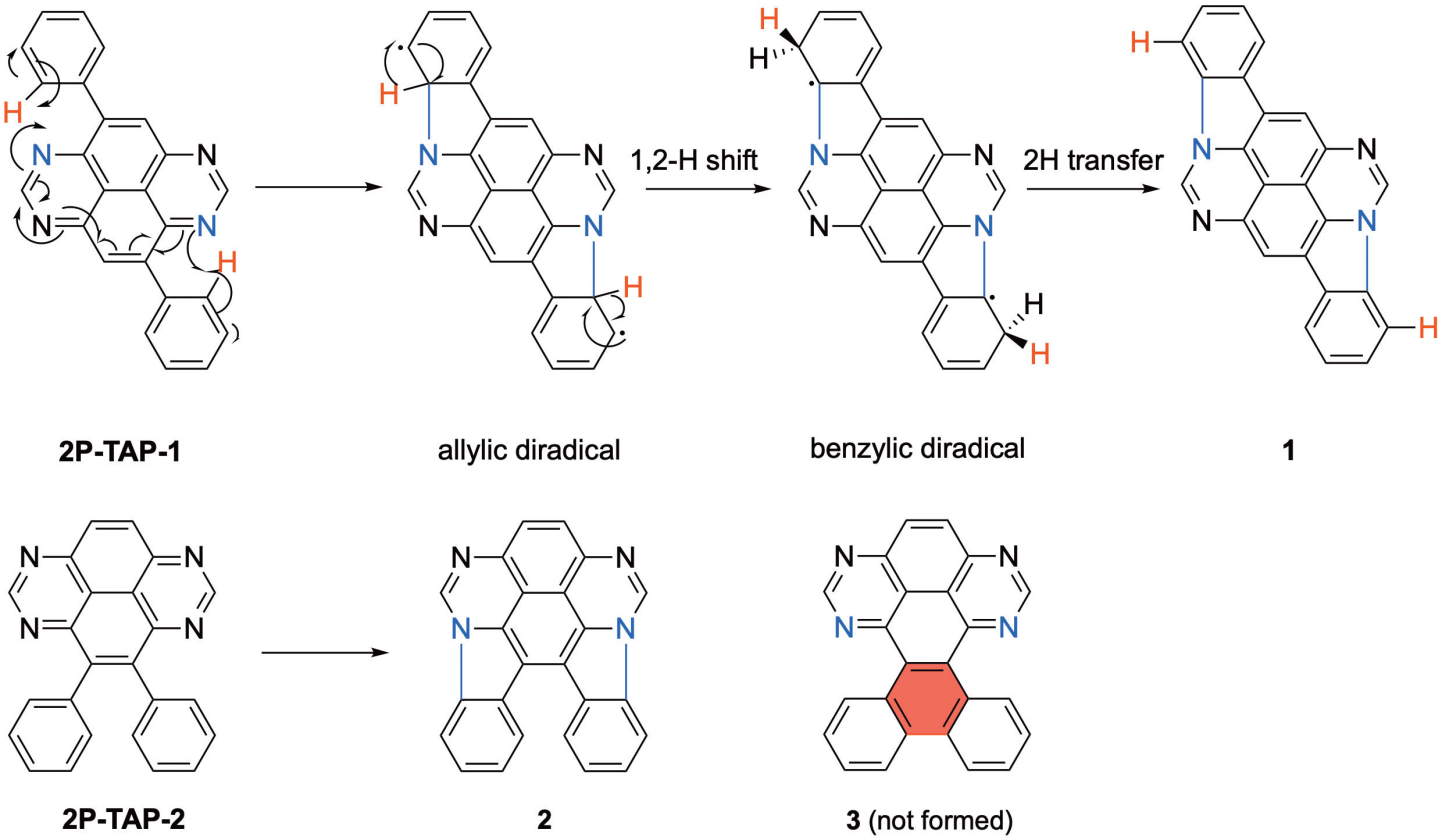
On‐surface synthesis of tetraazadiindenopyrene derivatives** 1** and **2** on Au(111). Tetraazaphenanthropyrene **3** was never formed.

The surface‐assisted CDH of **2P‐TAP‐1** and **2P‐TAP‐2** leads to the formation of two C─N bonds between the phenyl substituent and the TAP core, resulting in the cyclization of two aromatic pyrrole units per molecule. Both products **1** and **2** are aromatic, which contributes to their enhanced thermodynamic stability. However, we shall also note that intramolecular CDH between two ortho‐phenyl substituents on the TAP core in **2P‐TAP‐2** could, in principle, lead to the formation of tetraazaphenanthropyrene **3** where a new benzene ring is fused to the existing phenyl and TAP moieties (Scheme 2 below). This transformation has never been observed experimentally, implying that pyrrole cyclization is energetically more favorable than carbon‐ring closure within the TAP skeleton.

**SCHEME 3:**
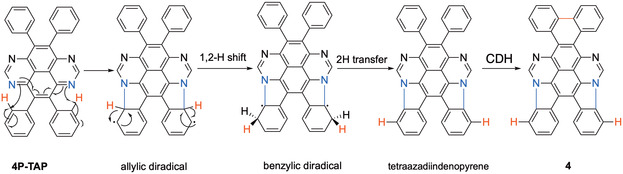
A plausible reaction mechanism for the transformation of **4P‐TAP** to **4** on Au(111).

Despite having the same number of phenyl end groups as **2P‐TAP‐1** and **2P‐TAP‐2**, annealing of **2P‐TAP‐3** at the moderate temperatures does not lead to CDH on Au(111) (Figure 2g). Only at a high annealing temperature of about 270°C do we observe the cyclization of a single pyrrole unit per molecule, with the second phenyl group remaining unreacted (Figure S3). We attribute this behavior to the loss of aromaticity in the TAP core, which would occur upon the formation of two pyrrole units and would likely prevent the further reaction of the other phenyl substituent in **2P‐TAP‐3**. Importantly, these observations highlight the critical influence of molecular geometry on the reaction pathway and activation energy barrier for surface‐assisted C─N bond formation.

As shown in Figure 2h, the CDH of **4P‐TAP** is asymmetric, as it produces two pyrrole units on one side of the TAP backbone (blue bonds in Figure 2l) and a single carbon‐ring closure between two phenyl groups on the other side (red bond). The product **4** adopts a “teddy‐bear” shape, and its aromaticity is retained after the reaction only if two pyrrole units are formed, but not more. This observation also implies a stepwise mechanism for the CDH of **4P‐TAP**, in which cyclization between phenyl groups becomes feasible only after the formation of two pyrrole units with the TAP core. Accordingly, we report the observation of an intermediate with two unreacted phenyl groups (Figure S2), which was presumably formed via a benzylic diradical pathway (Scheme 3).

### 2.3 Electronic Properties of the Teddy‐Bear Shaped Molecule **4**

**FIGURE 3:**
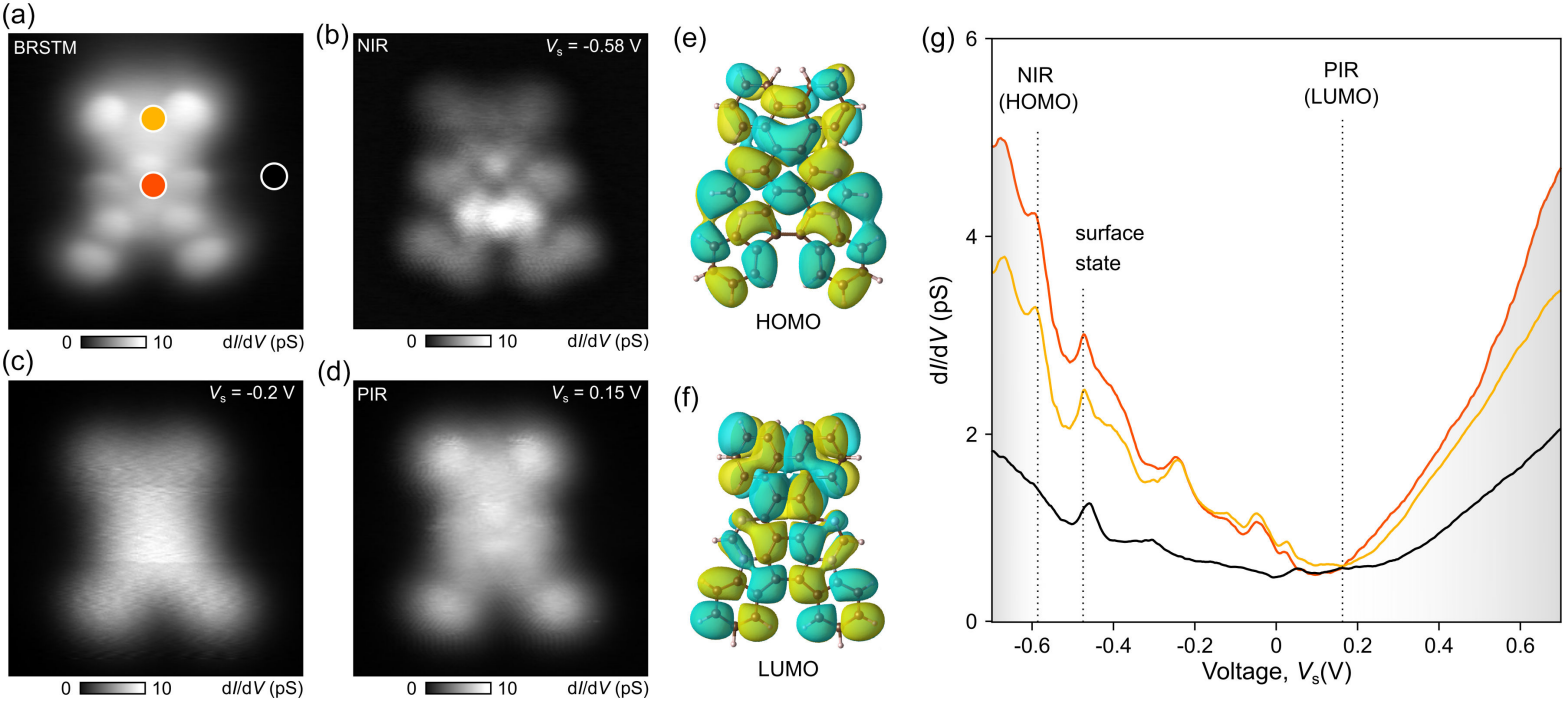
Electronic properties of the “teddy‐bear” molecule **4**. (a) Bond‐resolved STM image (voltage *V*  = −1 mV, tunneling current *I* = 10 pA, A_mod_ = 6 mV). (b–d) Constant‐height dI/dV maps of **4** at the PIR resonance, gap, and NIR resonances, respectively. (e,f) DFT orbitals of HOMO and LUMO of **4** in gas phase. (g) dI/dV point‐spectra extracted at the red, yellow, and black dots of a. PIR and NIR resonances are located at 0.15 and −0.58 V, respectively.

Differential conductance (dI/dV) point‐spectra acquired above the TAP backbone (red and yellow dots in the bond‐resolved STM image of Figure 3a) reveal two characteristic peaks associated with positive (PIR) and negative (NIR) ion resonances at sample voltages of V_s_ = −0.56 and 0.15 V, respectively (Figure 3g). We attribute them to the highest occupied (HOMO) and the lowest unoccupied molecular orbitals (LUMO), establishing a gap for the “teddy‐bear” molecule **4** of approximately 0.75 eV. The corresponding dI/dV maps recorded at these energies (Figure 3b,d) show the spatial distribution of the local density of states (LDOS) across the surface of the adsorbed molecule. Figure 3a reveals dot‐like states at the center of tetraazadiindenopyrene units, whereas the DOS at the phenanthrene core is less pronounced. For the LUMO energy, the DOS is more homogeneously distributed over the molecule with a nodal plane located at the center of the tetraazapyrene core. The calculated molecular orbitals for the free‐standing **4** molecule (Figure 3e,f) align well with these experimental observations.

### 2.4 Reaction Mechanism Investigated via DFT Calculations

**FIGURE 4:**
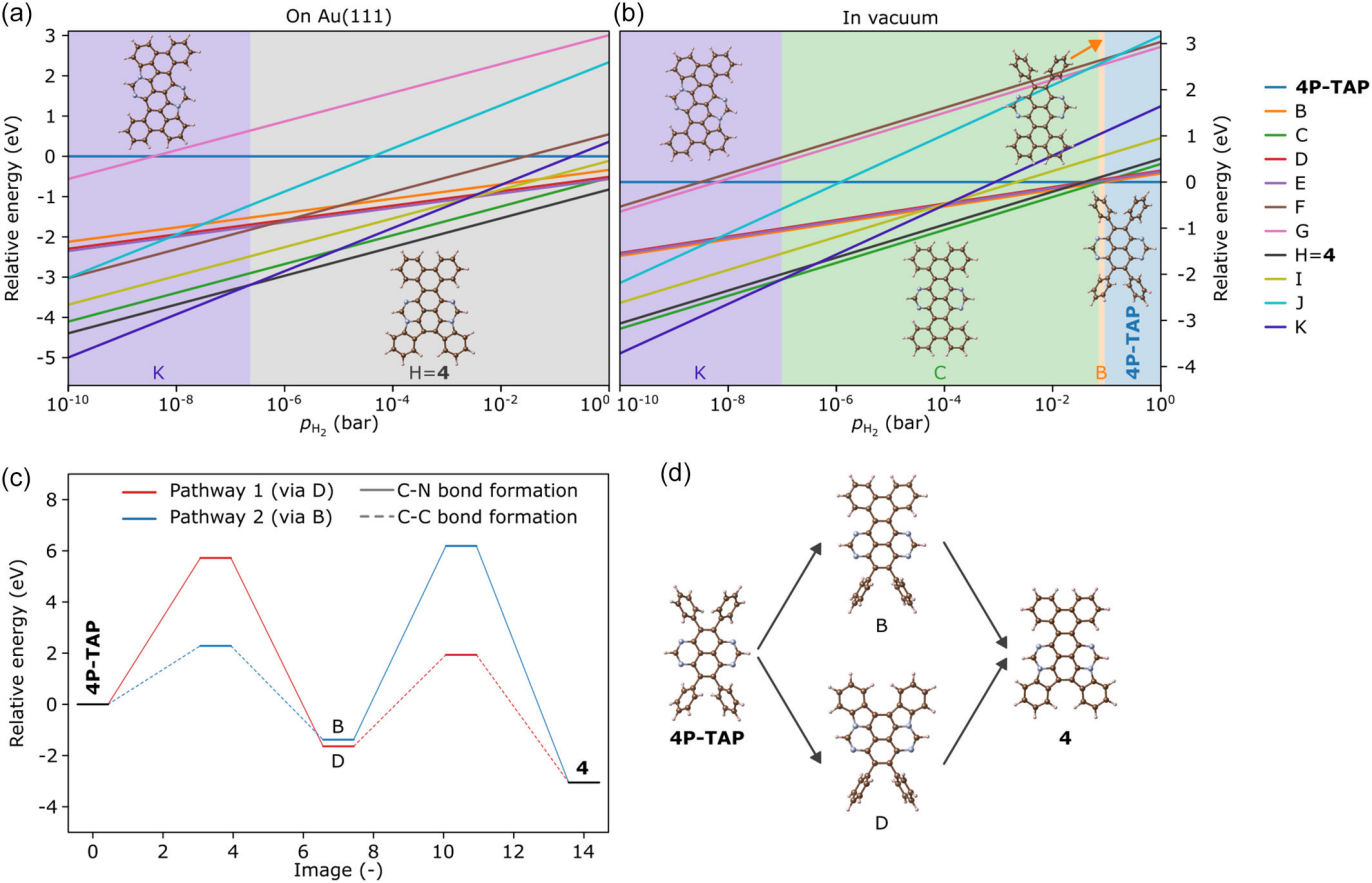
Relative stability of different cyclized potential products (B‐K) of **4P‐TAP** at 450 K as a function of the hydrogen partial pressure on the Au(111) surface (a), and in vacuum (b). (c,d) Relative energies of the transformation of **4P‐TAP** to **4** via two different pathways shown in d.

Initially, we screen the thermodynamic stability of differently cyclized potential products of **4P‐TAP** (Figure S4 for their structures) using DFT calculations. As the number of hydrogen atoms differs for these products, the analysis needs to be performed as a function of the hydrogen partial pressure. As shown in Figure 4a, we find that on the surface, the cyclized product H (**4**) is the most stable across a large range of hydrogen partial pressures, only replaced by a less hydrogen‐rich product K at very low partial pressures. This implies that compound **4** is stabilized on the surface compared to **4P‐TAP**. This is in stark contrast to vacuum (Figure 4b), where **4P‐TAP** is stable at ambient conditions and will be replaced by cyclized products B and C at lower hydrogen partial pressure. The surface, therefore, plays a crucial role in directing the reaction of **4P‐TAP** to compound **4**.

On Au(111), we next investigated the kinetic barriers for the transformation of **4P‐TAP** to compound **4** via nudged elastic band calculations. The transformation could occur via the intermediate D, starting with C─N bond formation, or via the intermediate B, starting with C─C bond formation. The results in Figure 4c show that in general, C─C bond formation is associated with smaller barriers, while also being less exothermic. More importantly, the barrier for C─N bond formation increases significantly after C─C bonds have been formed in B, resulting in a larger overall barrier for the blue pathway (via the intermediate B) compared to the red pathway (via the intermediate D). A complete transformation to the thermodynamically most stable compound **4** is thus more likely following the red pathway (C─N bond formation followed by C─C bond formation).

The CDH process thus likely begins with the formation of an allylic diradical transition state, akin to the C─N bond formation previously reported in tetraazateranthene [28], as illustrated in Scheme 3. This state then undergoes a rapid suprafacial [1, 2] hydrogen shift (red H atoms), resulting in a stabilized benzylic diradical intermediate state. The two hydrogen atoms located closest to the substrate (black H atoms) are then transferred to the Au(111) surface almost barrierless (aligned with the experimental conditions, *T* < 100°C), leading to the intermediate tetraazadiindenopyrene (Figure S2). This tetraazadiindenopyrene intermediate then undergoes further CDH, forming one C─C bond in a well‐established manner to produce the “teddy‐bear” molecule **4**. This study represents the first observation of stepwise covalent C─N and C─C bond formation with high selectivity, leading exclusively to the formation of compound **4**, in agreement with the experimental data of Figure 2.

To gain deeper insights into the observed high reaction selectivity, it is essential to understand why the two transition states, allylic diradical and benzylic diradical, are not formed preferentially along the diagonal direction, as depicted in Scheme 2. For this purpose, we discuss here the C─N single bond formation via surface‐assisted thermal CDH for **2P‐TAP‐1**, **2P‐TAP‐2,** and **2P‐TAP‐3**.

On Au(111), **2P‐TAP‐1** undergoes gradual cyclization, leading to a mixture of unreacted starting material, partially cyclized intermediates, and the fully transformed product **1**. In contrast, **2P‐TAP‐2** is completely transformed into tetraazadiindenopyrene **2** even under gentle annealing conditions. This significant difference indicates that the activation energy required to form the allylic diradical transition state, in which the two phenyl substituents are positioned diagonally opposite each other across the TAP core, is much higher than the energy required to form a transition state in which the phenyl groups are located on the same side of the TAP core. Interestingly, one of the phenyl groups in **2P‐TAP‐3** can participate in the formation of diradical transition states (with two of the eight possible resonance forms shown in Scheme S1), while the other cannot. Consequently, the associated activation energy is significantly higher than that of the isomers **2P‐TAP‐1** and **2P‐TAP‐2**. This is consistent with the aforementioned experimental observations: in **2P‐TAP‐3**, only one pyrrole unit per molecule cyclizes at 270°C (Figure S3), compared to two pyrrole units per molecule in **2P‐TAP‐1** and **2P‐TAP‐2** at 200°C. These findings highlight the critical influence of molecular geometry on the reaction pathway and activation energy barrier for surface‐assisted C─N bond formation.

These experimental findings are further supported by DFT calculations (Figure S5) that show product **1** to be thermodynamically most stable for **2P‐TAP‐1** for hydrogen partial pressures below ambient, as they occur in a STM/AFM chamber. For **2P‐TAP‐2**, our calculations predict product **2** to be most stable, except for very low hydrogen partial pressures, while product **3** is never formed. For **2P‐TAP‐3**, we predict the unreacted form to persist to smaller hydrogen partial pressures compared to **2P‐TAP‐1**, forming first one and then two C─N bonds, as the partial pressure is reduced.

## 3 Conclusion

We have developed an efficient and novel synthetic protocol for the cascade bromination of the TAP core, which enables the preparation of the corresponding mono‐, di‐, and tri‐phenyl TAP derivatives via a one‐pot reaction with phenylboronic acid. In particular, three diphenyl TAP isomers were isolated, enabling us to elucidate the role of molecular geometry and aromaticity in dictating the selective pyrrole cyclization of phenyl‐substituted TAP derivatives on Au(111). STM and AFM images have been used to visualize the chemical structures of the newly formed tetraazadiindenopyrene derivatives, unambiguously showing C─N bond formation over the well‐established C─C bond formation. Furthermore, DFT calculations have provided mechanistic insight into the sequential C─N and C─C cyclization processes. Together, these findings clarify how oxidative C─N bonds form on metal surfaces and deliver a valuable synthetic approach for precisely incorporating heteroatoms into extended π‐systems. This work paves the way for designing next‐generation N‐doped nanographenes.

## Supporting Information

Additional supporting information can be found online in the Supporting Information section. The authors have cited additional references within the Supporting Information [48, 49, 50, 51, 52, 53].

## Funding

This study was supported by Schweizerischer Nationalfonds zur Förderung der Wissenschaftlichen Forschung (200021 204053, 200020 188445 and CRSII5 213533), Werner Siemens‐Stiftung, Swiss Nanoscience Institute, H2020 European Research Council (834402).

## Conflicts of Interest

The authors declare no conflicts of interest.

## Supporting information

Supplementary Material

## Data Availability

The data that supports the findings of this study are available in the supplementary material of this article.
