# Supplementary material for: First Access to Tetraazadiindenopyrenes via Selective Pyrrole Cyclization of Phenyl‐Substituted Tetraazapyrene Derivatives on Au(111)
Source: Small Sci. 2026 May 8;6(5):e70288. doi: 10.1002/smsc.70288 (PMC13155248; doi:10.1002/smsc.70288)
Supplement: Supplementary file 1 — Supplementary Material [file SMSC-6-e70288-s001.pdf]

Supplementary information

First Access to Tetraazadiindenopyrenes via Selective Pyrrole Cyclization of Phenyl-Substituted Tetraazapyrene Derivatives on Au(111)

Isabelle Kolly, Gema Navarro-Marín, Robert Häner, Silvio Decurtins, Ernst Meyer, Ulrich Aschauer,* Rémy Pawlak* and Shi-Xia Liu*

Experimental Section

All reagents and solvents were purchased from commercial sources and used without further purification. TBTAP and TAP were prepared as described in the literature.[39-41] Chemicals used for the synthesis of the compounds were purchased from commercial suppliers (Sigma-Aldrich, TCI or Alfa Aesar). ^{1}H and ^{13}C NMR spectra were recorded on a Bruker Avance 300 or 400 spectrometer at 300 MHz and 75 MHz or 101 MHz, respectively. Chemical shifts are reported in parts per million (ppm) and are referenced to the residual solvent peak ($CDCl_3$, δ ^{1}H = 7.26 ppm, δ ^{13}C = 77.16 ppm). The following abbreviations were used s (singlet), d (doublet), t (triplet) and m (multiplet). Mass spectra were measured by the Analytical Research and Services (ARS) of the University of Bern, Switzerland, on a Thermo Fisher LTQ Orbitrap XL using Nano Electrospray Ionization (NSI).

Synthesis of tetraphenyl-TAP (**4P-TAP**)

A modified protocol from previous literature[41] was followed. A dried round neck flask was loaded with TBTAP (50 mg, 0.096 mmol), phenylboronic acid (93 mg, 0.767 mmol), cesium carbonate (312 mg, 0.958 mmol) and Pd(dppf)Cl_2 (14 mg, 0.019 mmol) and then evacuated and refilled with argon three times. Dry 1,4-dioxane (17 mL) was added and the reaction mixture was stirred at 100 °C for 48 h under inert conditions. After completion, the reaction mixture was cooled down to room temperature, and the solvent was evaporated. The residue was suspended in water (50 mL) and extracted with chloroform three times (3× 30 mL). The combined organic phase was washed with brine (3× 40 mL), water (3× 50 mL) and dried over sodium sulfate. After removing the solvent, the resultant crude product was purified by flash column chromatography (silica gel, CH_2Cl_2/heptane 1:1, v/v) to give the pure compound as a yellow solid (18 mg, 35%). ^{1}H NMR (300 MHz, $CDCl_3$) δ 10.04 (s, 2H), 7.41 – 7.33 (m, 20H). ^{13}C NMR (75 MHz, $CDCl_3$) δ 157.78, 152.40, 146.25, 135.50, 131.42, 127.95, 127.75. HR-MS (ESI, positive): *m/z* calc. for $[C_{36}H_{22}N_4H]^+$ 511.1917; found: 511.1898.

One-pot synthesis of mono-, three di- and tri-substituted TAPs:

IBr (100 mg, 0.485 mmol) was added to a solution of TAP (50 mg, 0.242 mmol) in CF_3SO_3H (6 mL). The reaction mixture was stirred at 80 °C overnight. After cooling down to room temperature, the reaction mixture was poured on ice, neutralized with Na_2CO_3 and extracted with $CHCl_3$ three times (3× 30 mL). The combined organic phase was washed with brine (3× 30 mL), water (3× 40 mL) and dried over sodium sulfate. The solvent was removed under reduced pressure, and the crude product was used for the next step without further purification. The dried crude product was loaded in an oven dried flask, followed by the addition of phenylboronic acid (150 mg, 1.229 mmol), cesium carbonate (448 mg, 1.374 mmol) and Pd(dppf)Cl_2 (20 mg, 0.027 mmol). The reaction mixture was evacuated and refilled with argon three times. Dry 1,4-dioxane (17 mL) was added and the reaction mixture was stirred at 100 °C for 24 h under inert conditions. After completion, the mixture was cooled down to

room temperature, and the solvent was evaporated. The residue was suspended in water (50 mL) and extracted with chloroform three times (3× 50 mL). The combined organic phase was washed with brine (3× 50 mL), water (3× 50 mL) and dried over sodium sulfate. After removing the solvent, the resultant crude product was purified by flash column chromatography on silica gel, eluting with a mixture of CH_2Cl_2 and EtOAc (gradient from 1:0 → 1:1, v/v) to give the different isomers as pure yellow/brownish solids (**3P-TAP**, 7 mg, 4%), (**2P-TAP-1**, 6 mg, 7%), (**2P-TAP-2**, 8 mg, 9%), (**2P-TAP-3**, 5 mg, 6%), (**1P-TAP**, 23 mg, 34%).

3P-TAP ^{1}H NMR (300 MHz, $CDCl_3$) δ 10.10 (s, 1H), 10.04 (s, 1H), 8.75 (s, 1H), 8.06 – 7.97 (m, 2H), 7.71 – 7.58 (m, 3H), 7.42 – 7.32 (m, 10H).^{13}C NMR (101 MHz, $CDCl_3$) δ 158.02, 157.45, 153.02, 152.28, 152.15, 151.70, 148.14, 146.45, 146.17, 136.29, 135.44, 135.30, 134.78, 131.43, 131.41, 130.77, 129.35, 128.64, 127.98, 127.75, 114.44, 113.55. HR-MS (ESI, positive): *m/z* calc. for $[C_{30}H_{18}N_4H]^+$ 435.1604; found: 435.1598.

2P-TAP-1 ^{1}H NMR (400 MHz, $CDCl_3$) δ 10.10 (s, 2H), 8.73 (s, 2H), 8.09 – 7.91 (m, 4H), 7.76 – 7.53 (m, 6H). ^{13}C NMR (101 MHz, $CDCl_3$) δ 157.68, 152.77, 151.60, 148.04, 136.07, 134.94, 130.75, 129.41, 128.65, 114.24. HR-MS (ESI, positive): *m/z* calc. for $[C_{24}H_{14}N_4H]^+$ 359.1291; found: 359.1290.

2P-TAP-2 ^{1}H NMR (300 MHz, $CDCl_3$) δ 10.05 (s, 2H), 8.74 (s, 2H), 7.48 – 7.29 (m, 10H). ^{13}C NMR (101 MHz, $CDCl_3$) δ 157.81, 152.70, 152.37, 146.29, 136.28, 135.24, 131.41, 128.01, 127.75, 113.91. HR-MS (ESI, positive): *m/z* calc. for $[C_{24}H_{14}N_4H]^+$ 359.1291; found: 359.1288.

2P-TAP-3 ^{1}H NMR (300 MHz, $CDCl_3$) δ 10.15 (s, 1H), 10.06 (s, 1H), 8.72 (s, 2H), 8.05 – 7.88 (m, 4H), 7.74 – 7.51 (m, 6H). ^{13}C NMR (101 MHz, $CDCl_3$) δ 158.25, 157.08, 152.27, 152.03, 148.43, 136.25, 134.70, 130.77, 129.37, 128.63, 115.11, 113.35. HR-MS (ESI, positive): *m/z* calc. for $[C_{24}H_{14}N_4H]^+$ 359.1291; found: 359.1290.

1P-TAP ^{1}H NMR (300 MHz, $CDCl_3$) δ 10.11 (s, 1H), 10.07 (s, 1H), 8.77 – 8.66 (m, 3H), 8.00-7.94 (m, 2H), 7.68 – 7.59 (m, 3H). ^{13}C NMR (101 MHz, $CDCl_3$) δ 158.18, 157.60, 153.07, 152.61, 152.44, 152.12, 148.41, 136.69, 136.32, 136.21, 134.89, 130.88, 129.57, 128.79, 114.76, 113.86. HR-MS (ESI, positive): *m/z* calc. for $[C_{18}H_{10}N_4H]^+$ 283.0975; found: 283.0978.

STM/AFM experiments

Sample preparation and characterization were performed in a low temperature STM/AFM operated at 4.7 K in ultra-high vacuum (≈ 3×10^{-10} mbar). The Au(111) substrate was purchased from Mateck GmbH and cleaned by successive cycles of Ar^+ ion sputtering and annealing at 450 °C. Molecules were sublimated from a Knudsen cell evaporator onto the gold surface held at room temperature, followed by annealing to induce the on-surface reactions. All measurements were carried out with a tungsten tip attached to a tuning fork in a qPlus sensor configuration, with resonance frequency f_0= 25.89 kHz, quality factor Q= 9042 and spring constant k≈ 1800 Nm^{-1}. CO molecules were dosed onto the cold sample to induce the formation of CO-decorated tips used for dI/dV maps and AFM imaging. All STM images were acquired in constant current mode, whereas the nc-AFM images were taken at constant tip-sample distances. Bias voltage (V_s) was applied to the tip with respect to the sample.

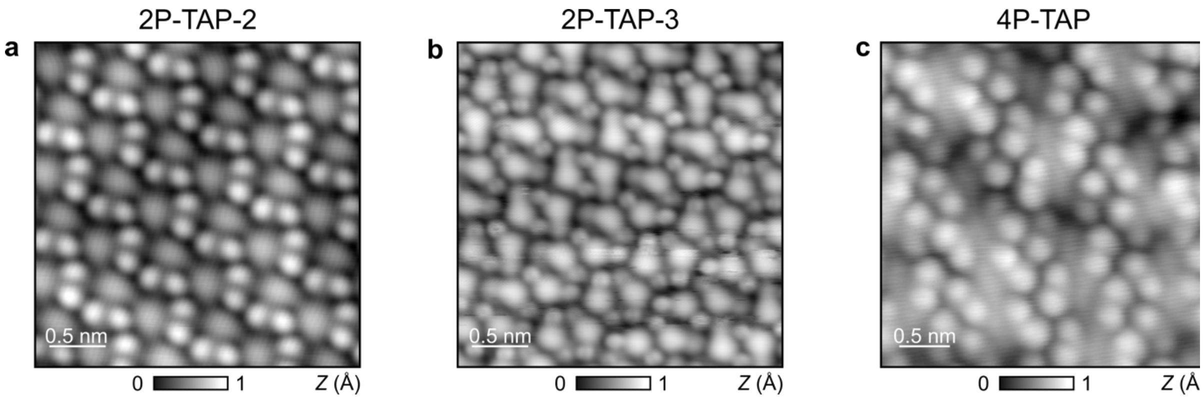

Figure S1: Supramolecular assemblies of **2P-TAP-2**, **2P-TAP-3** and **4P-TAP**.

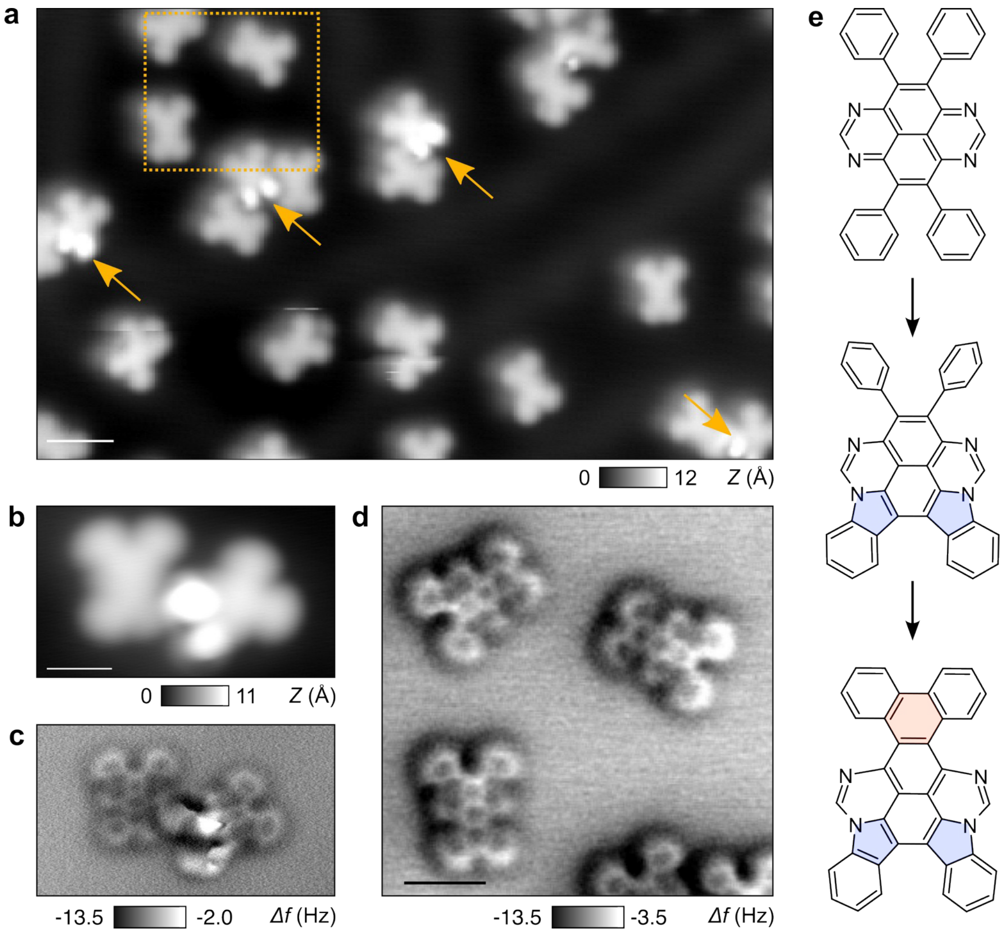

Figure S2: Identification of a tetraazadiindenopyrene intermediate. **a**, STM image of **4P-TAP** molecules on Au(111) upon thermal annealing at about 475 K, (*I* = 1 pA, *V* = 50 mV). Most molecules have a crossed shape while few of them shows bright protrusions as marked by orange arrows. Scale bar is 2 nm. **b**, Close-up STM image of a protruded molecule and **c**, The corresponding AFM image acquired with a CO-terminated tip. Bright spots correspond to unreacted phenyl rings on one side of the molecules. Scale bar is 1 nm. **d**, Representative AFM image of the cross-shape molecules acquired in the yellow dashed rectangle of **a**. Scale bar is 1 nm. **e**, Scheme of the chemical transformation of **4P-TAP** to **4**. An asymmetric cyclodehydrogenation reaction occurs in two steps, consisting of the formation of two pyrrole units (blue) on one side of the molecule followed by a ring closure (red) between the remaining phenyls on the other side.

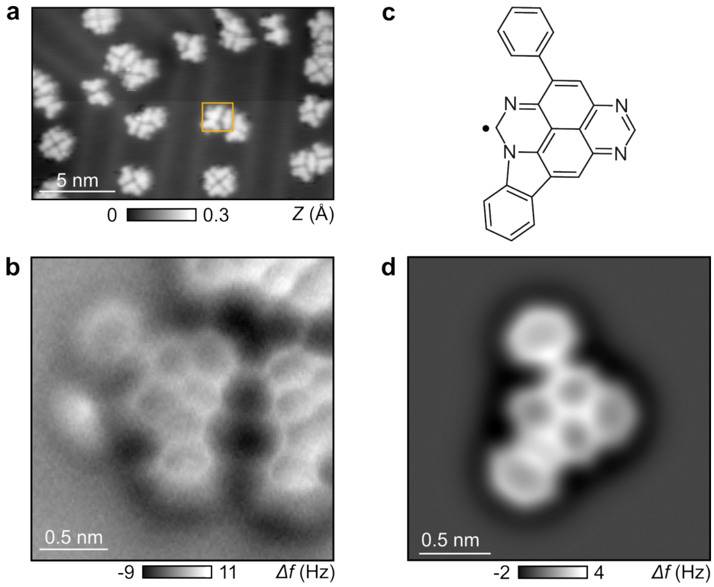

Figure S3: Reaction of **2P-TAP-3** at high temperature. **a,** STM image of molecules on Au(111) upon thermal annealing at about 500 K, (I = 1 pA, V = 50 mV). **b**, Constant-height AFM images of the reacted products (oscillation amplitude A_{OSC} = 50 pm). **c**, Interpreted chemical structure of the reacted products based on the AFM image. **d**, Simulated AFM images using the probe-particle model model.

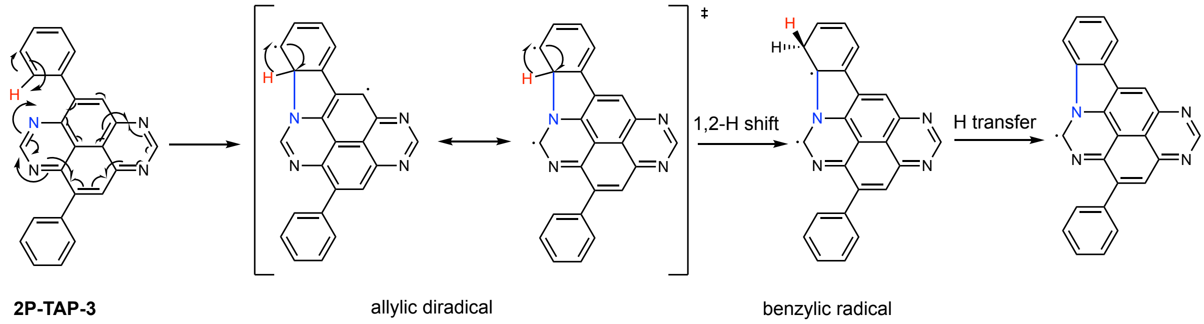

Scheme S1: A plausible reaction mechanism of **2P-TAP-3** on Au(111).

DFT calculations

DFT calculations were performed within the Gaussian plane wave method, [48] as implemented in the Quickstep[49] module of CP2K.[50] We used the PBE density functional[51] together with a D3 dispersion correction,[52] a double-zeta molecularly optimized basis set[53] and Goedecker-Teter-Hutter pseudopotentials.[53] The Au(111) surface was modeled as a 4-layer thick slab in a supercell with a 23.07 Å lattice parameter. The hydrogen chemical potential at a given partial pressure was computed as $\mu_H = \frac{1}{2}E_{H_2} + k_B T ln\frac{p_H}{p_0}$.

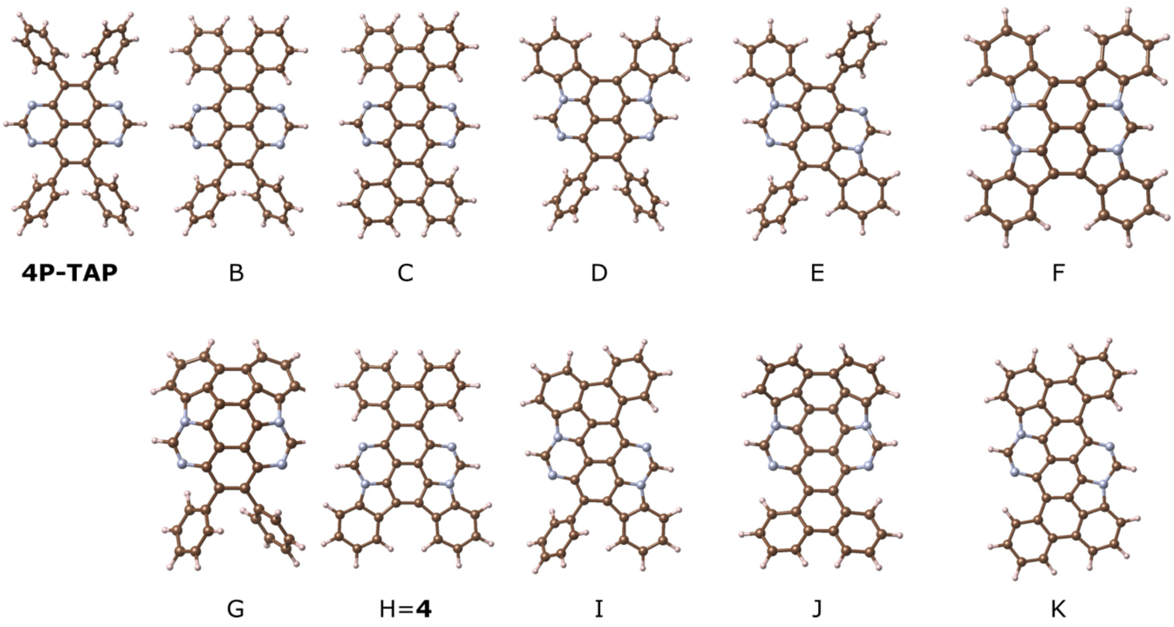

Fig. S4: Different dehydrogenated and cyclized potential products (B-K) of **4P-TAP** considered in DFT calculations.

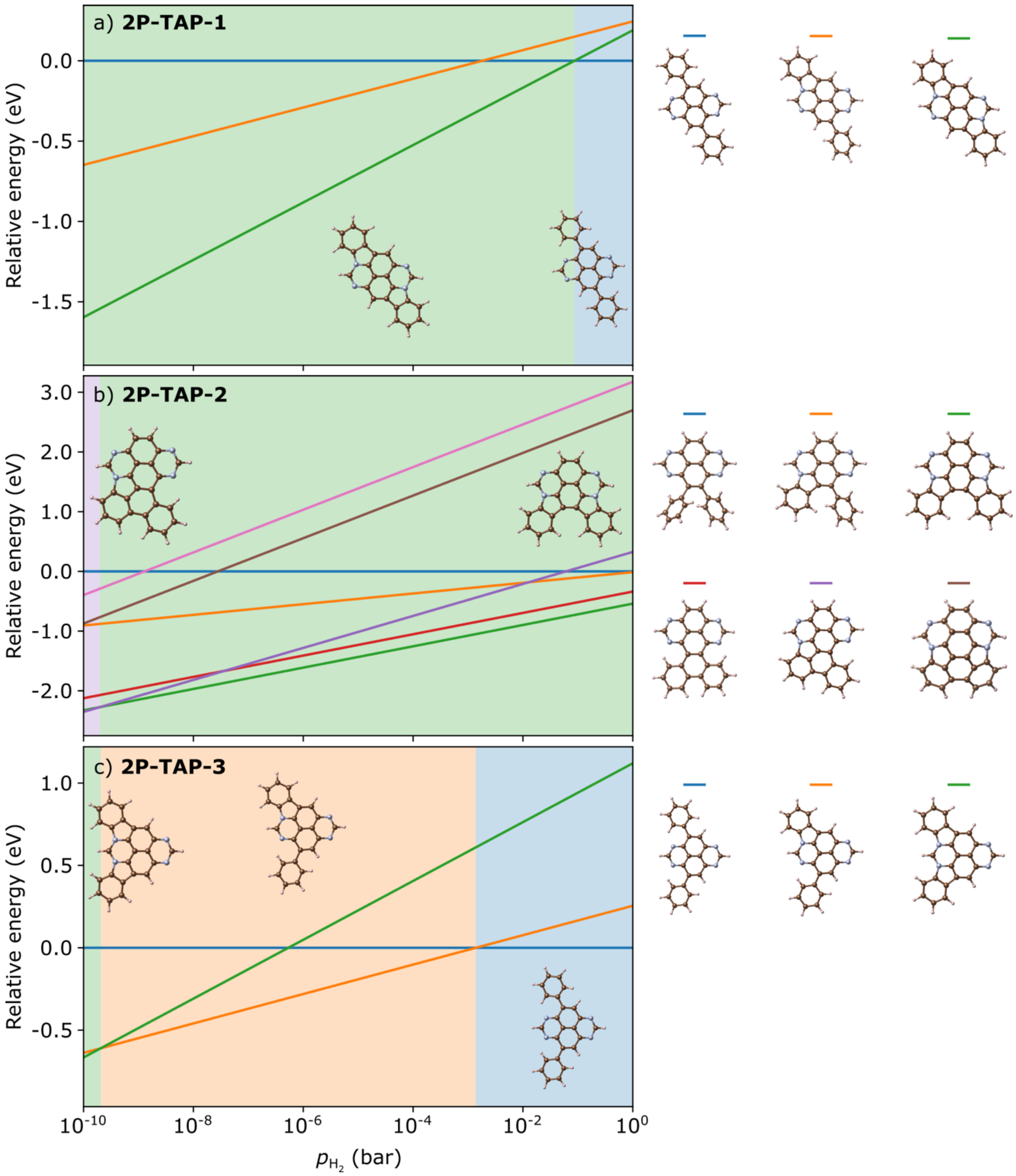

Fig. S5: Relative energy as a function of the hydrogen partial pressure for a) **2P-TAP-1**, b) **2P-TAP-2** and c) **2P-TAP-3**. The considered potential products are shown on the right.

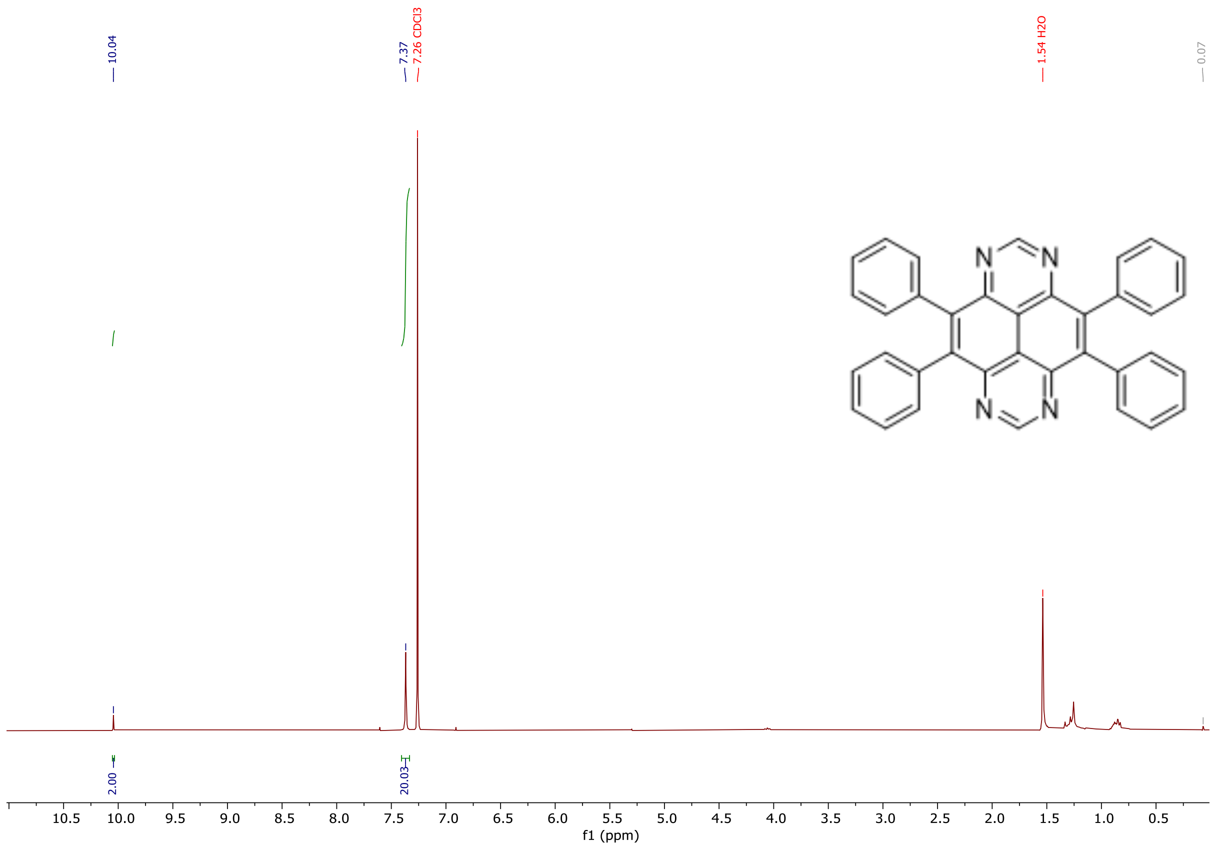

Figure S6: ^{1}H NMR spectrum of **4P-TAP**.

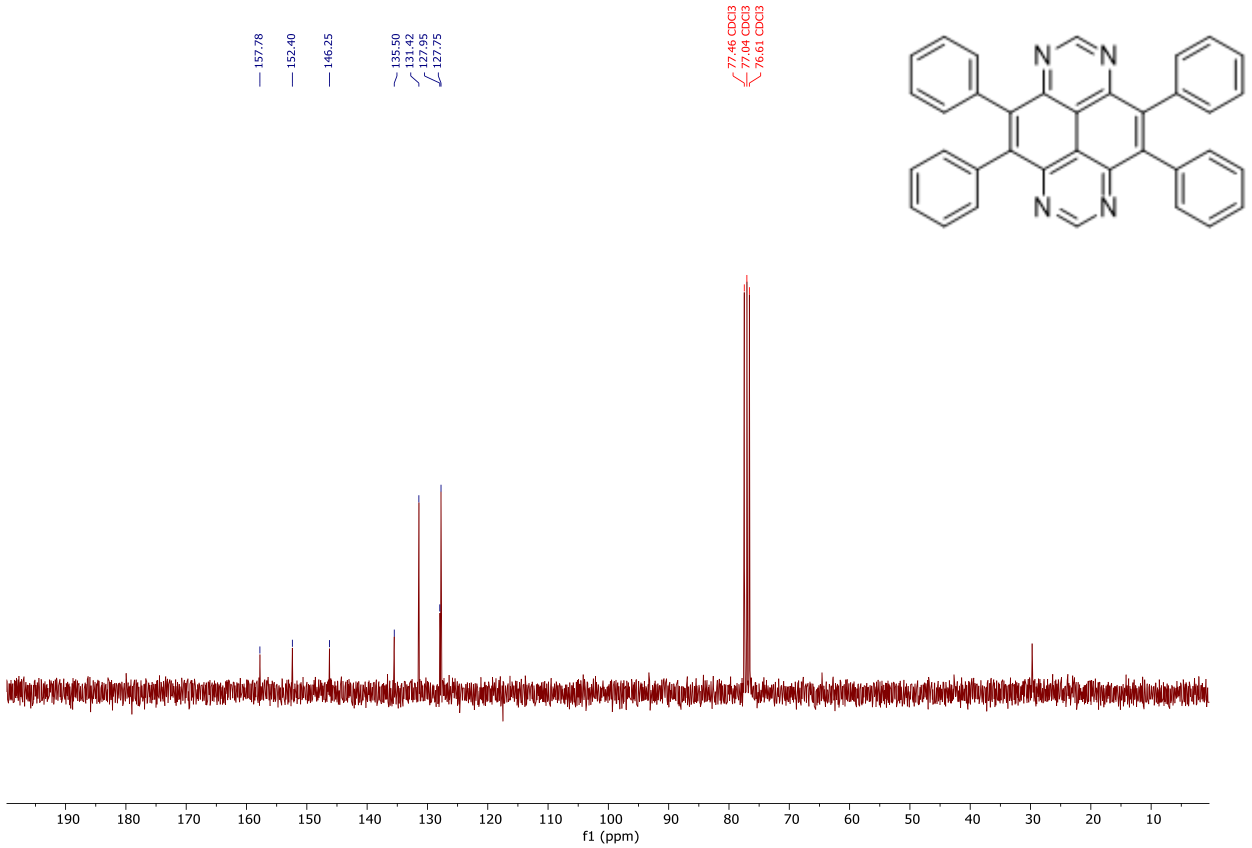

Figure S7: ^{13}C NMR spectrum of **4P-TAP**.

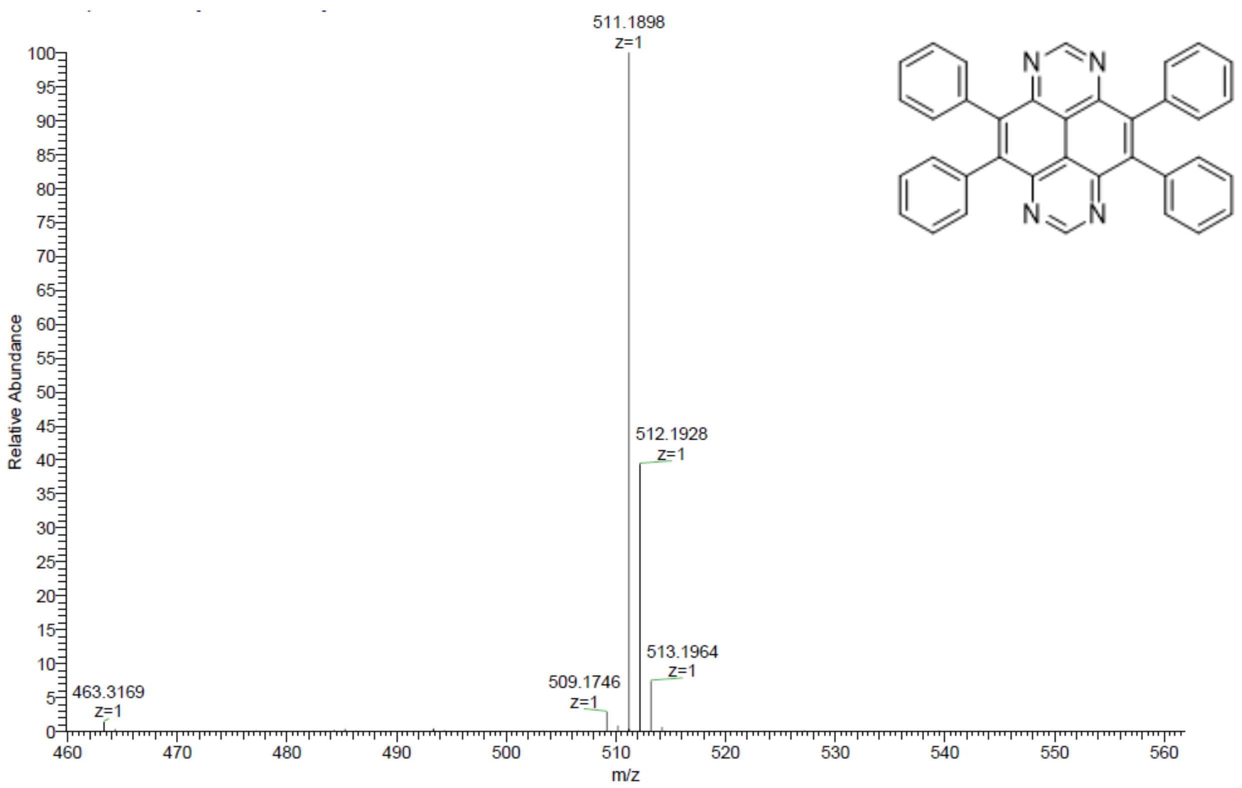

Figure S8: HR-MS (ESI, positive) spectrum of **4P-TAP**.

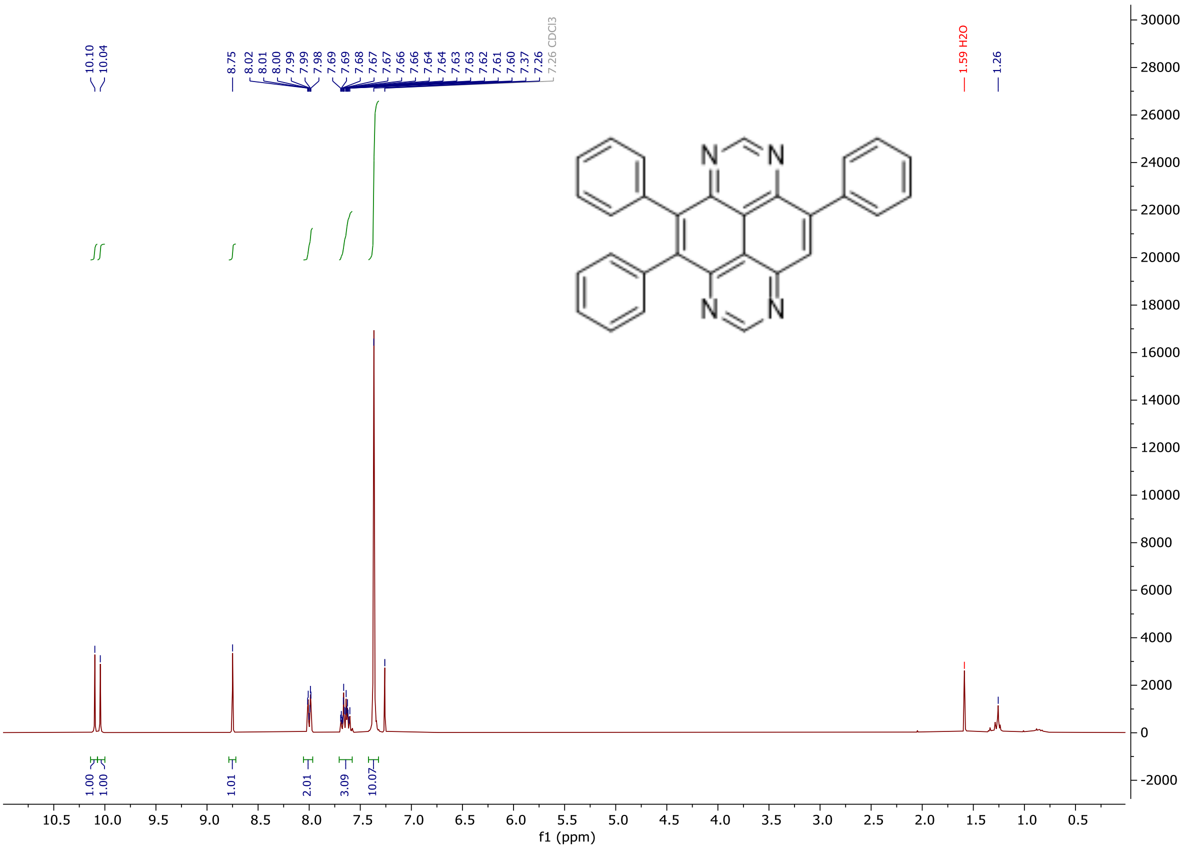

Figure S9: ^{1}H NMR spectrum of **3P-TAP**.

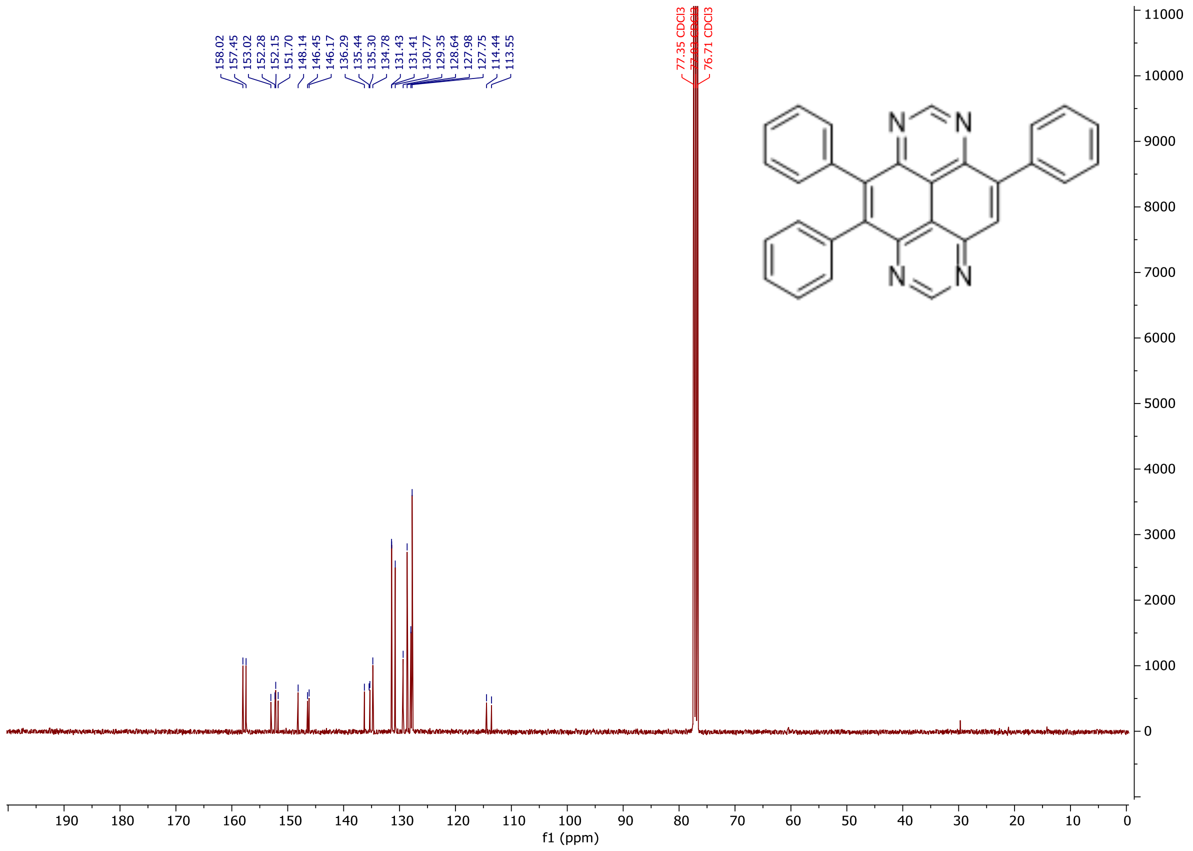

Figure S10: ^{13}C NMR spectrum of **3P-TAP**.

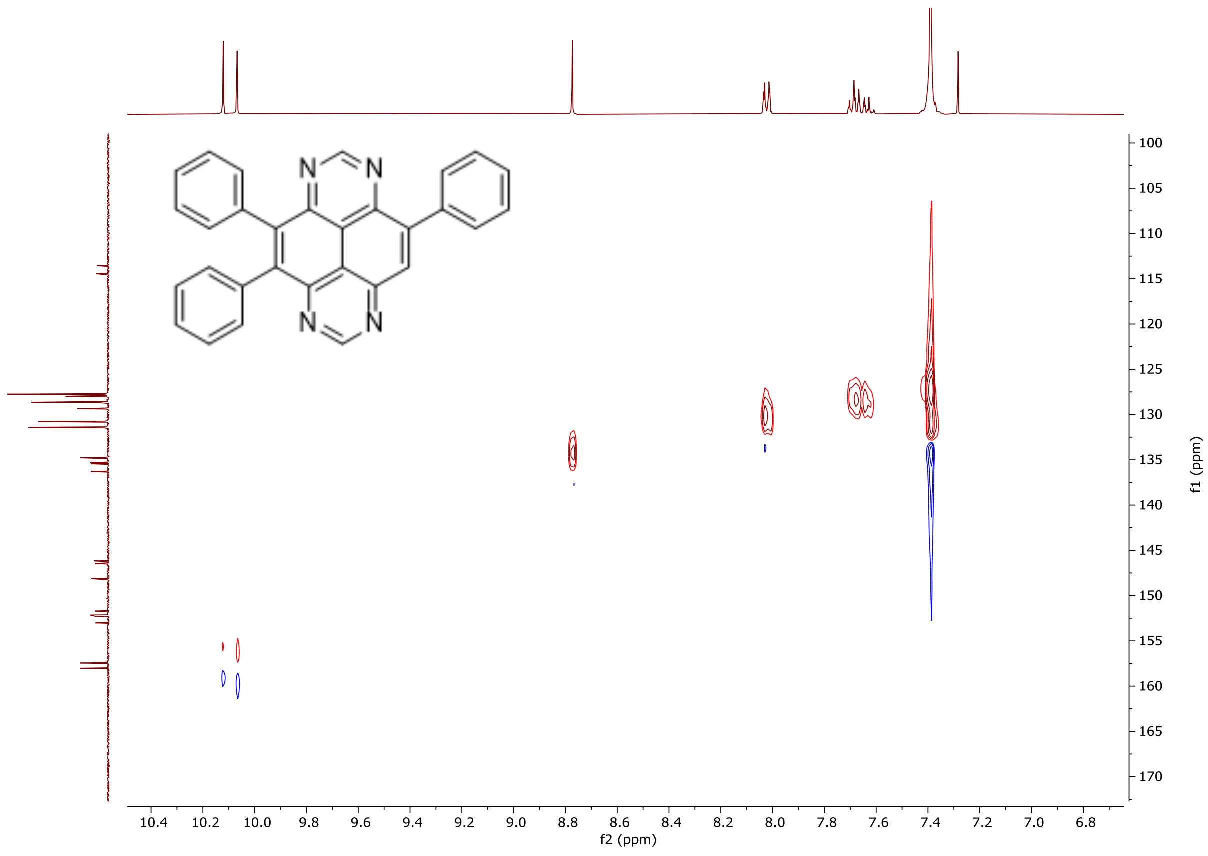

Figure S11: ^{13}C/^{1}H-HSQC NMR spectrum of **3P-TAP**.

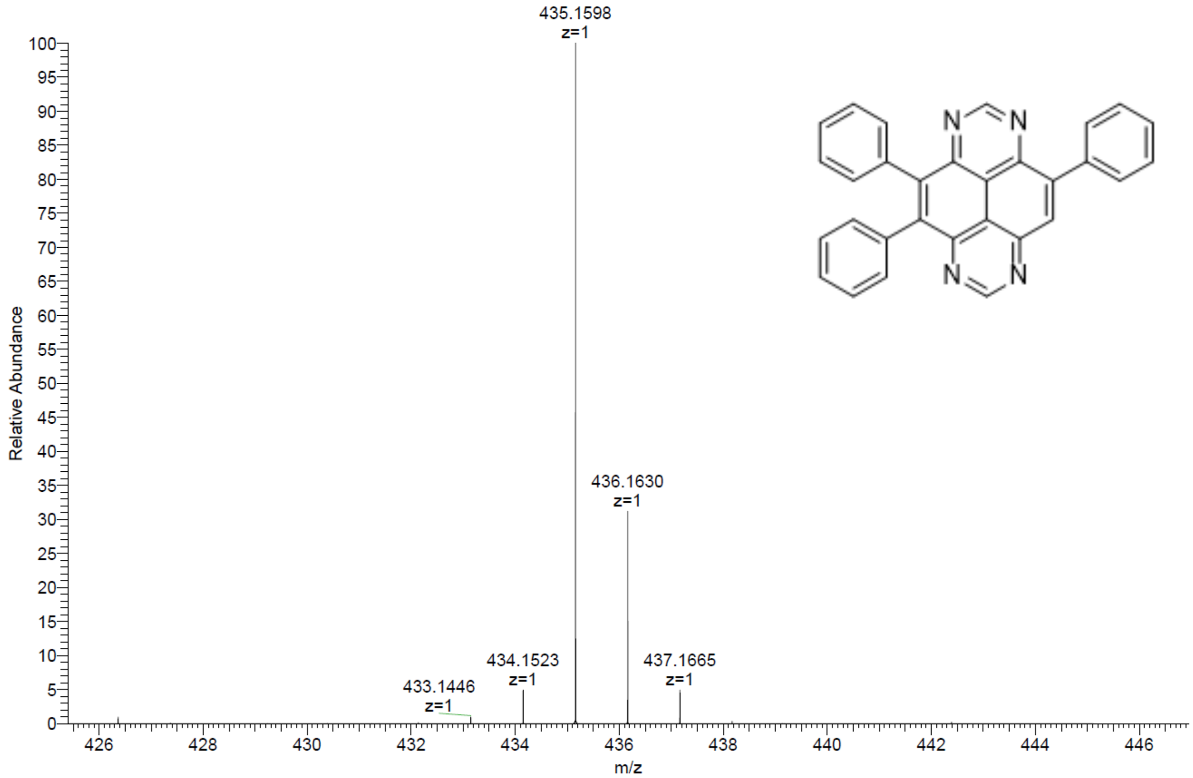

Figure S12: HR-MS (ESI, positive) spectrum of **3P-TAP**.

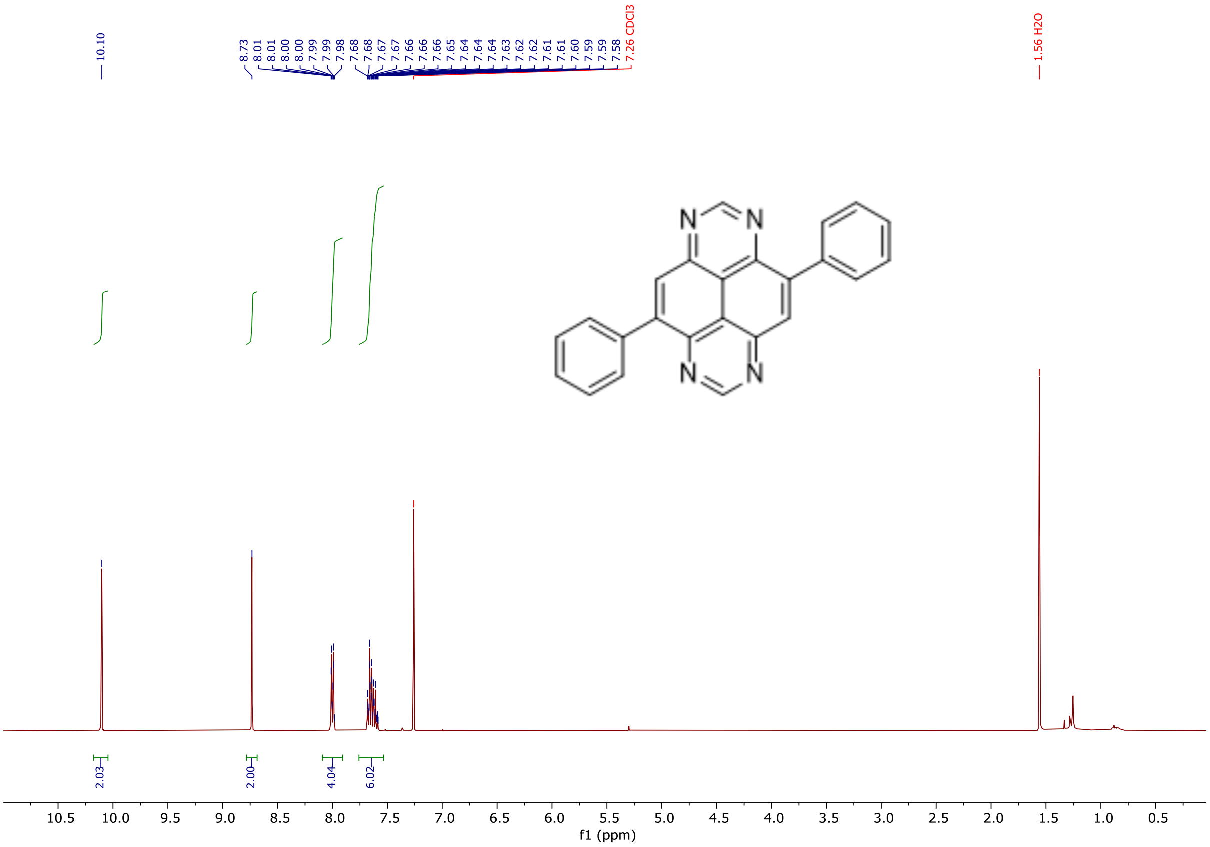

Figure S13: ^{1}H NMR spectrum of **2P-TAP-1**.

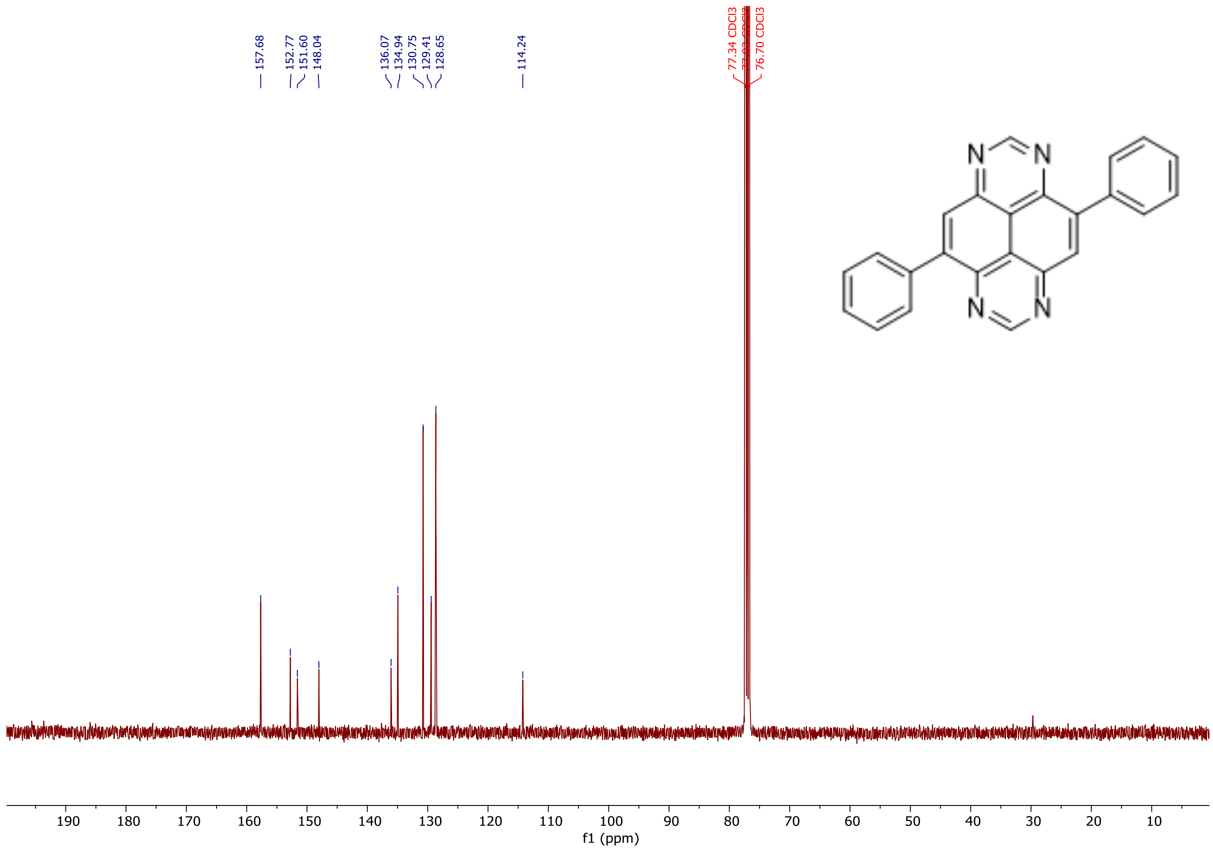

Figure S14: ^{13}C NMR spectrum of **2P-TAP-1**.

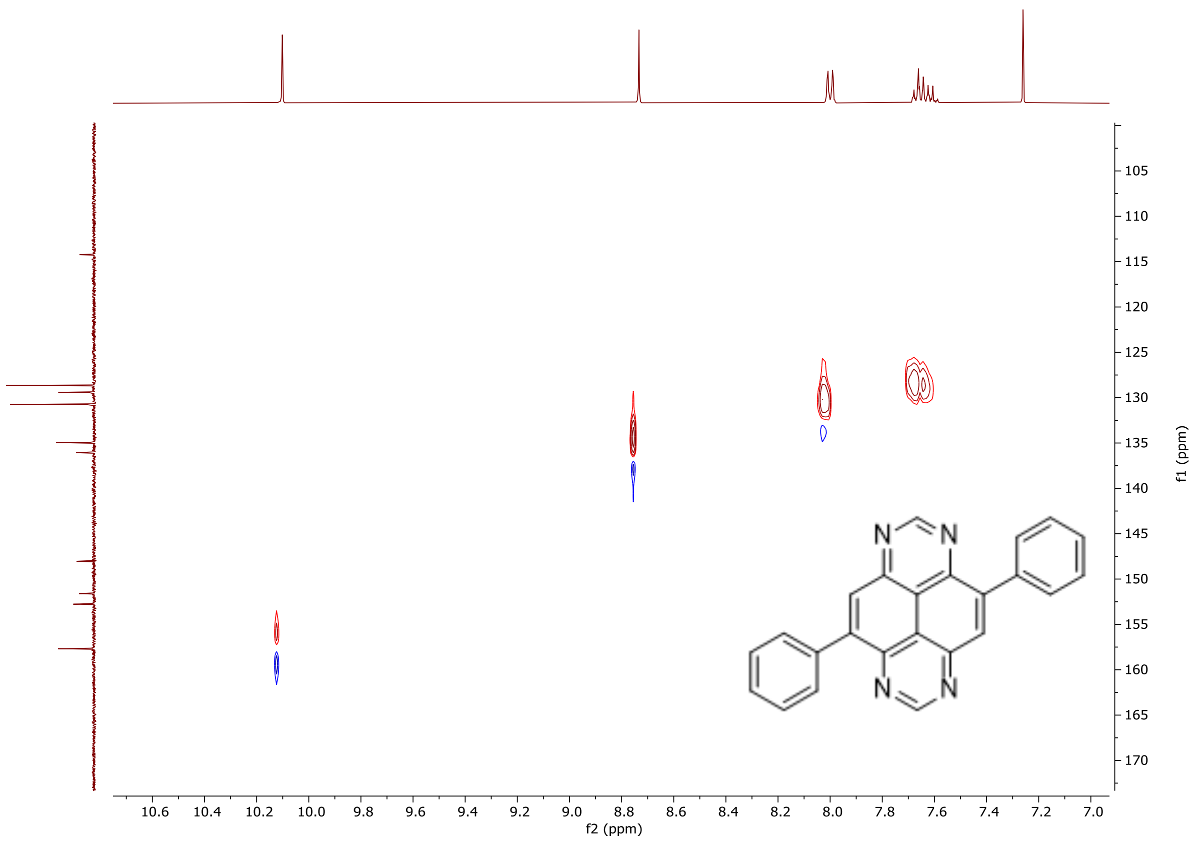

Figure S15: ^{13}C/^{1}H-HSQC NMR spectrum of **2P-TAP-1**.

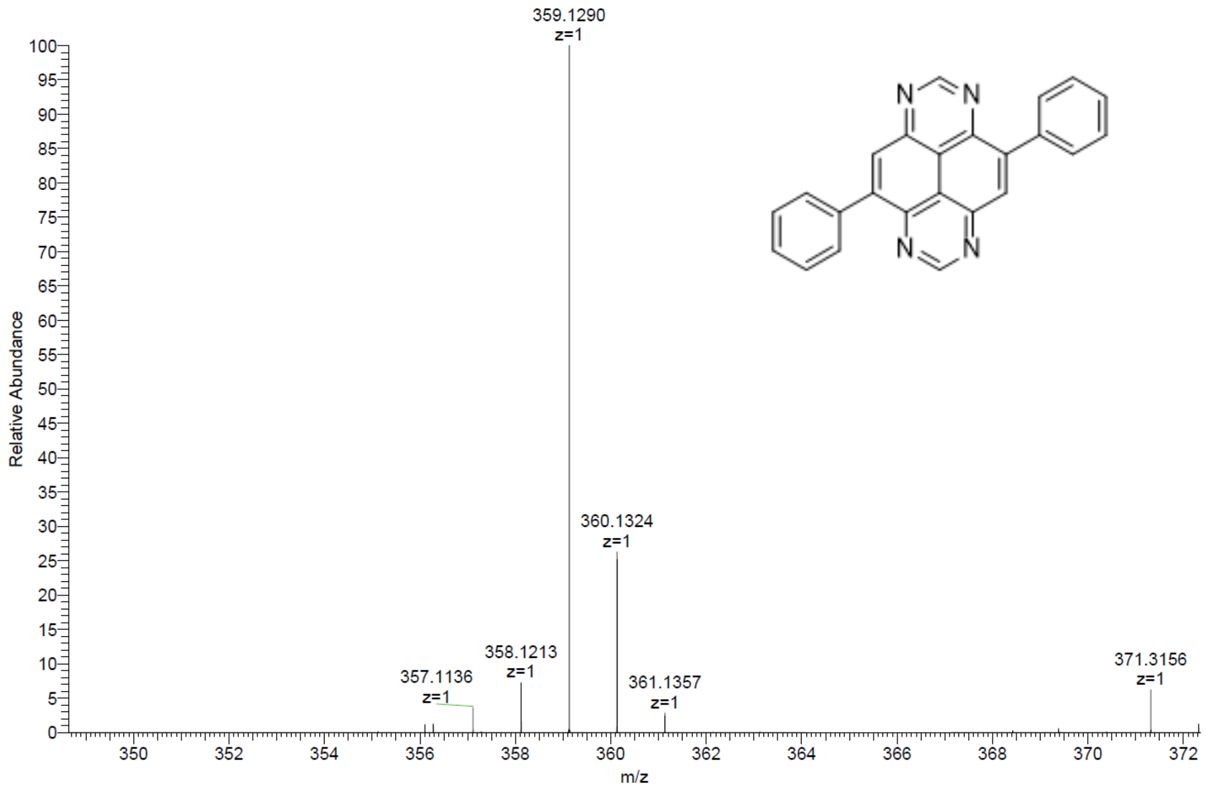

Figure S16: HR-MS (ESI, positive) spectrum of **2P-TAP-1**.

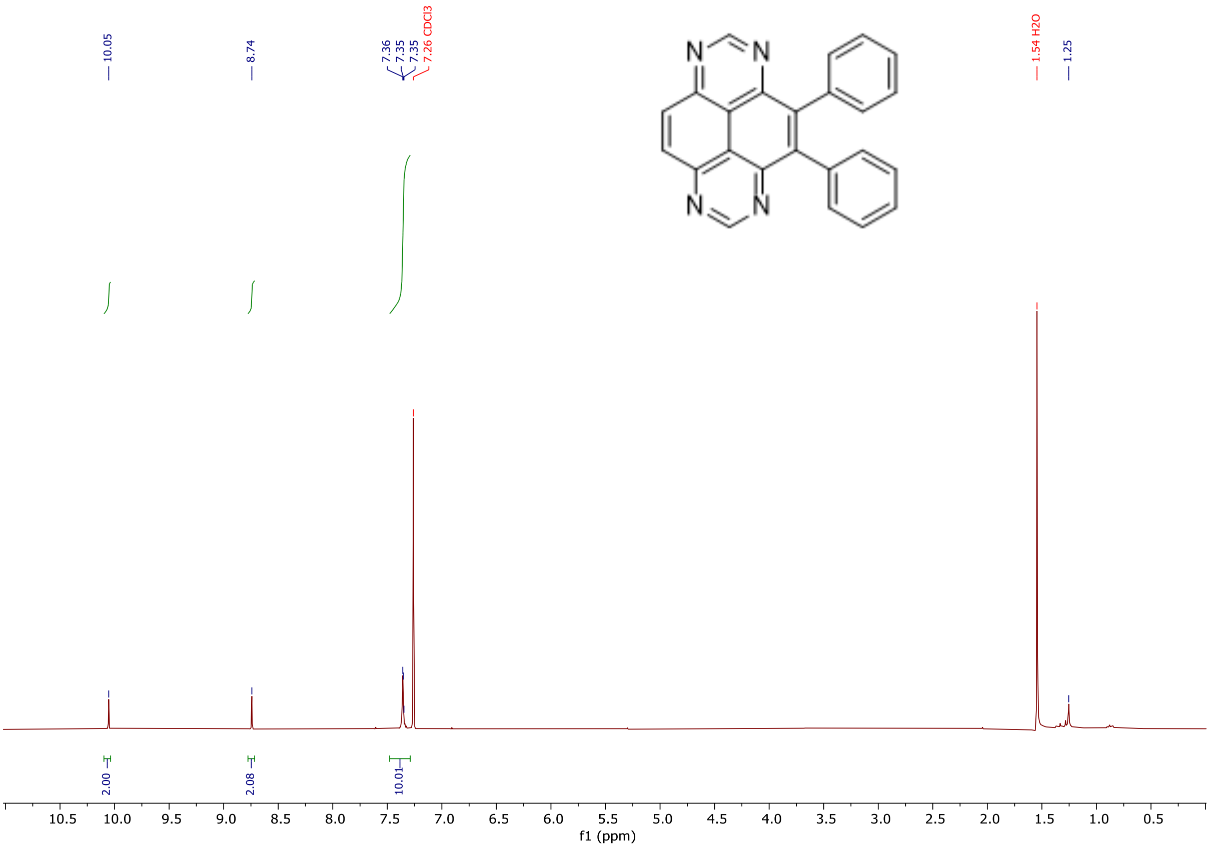

Figure S17: ^{1}H NMR spectrum of **2P-TAP-2**.

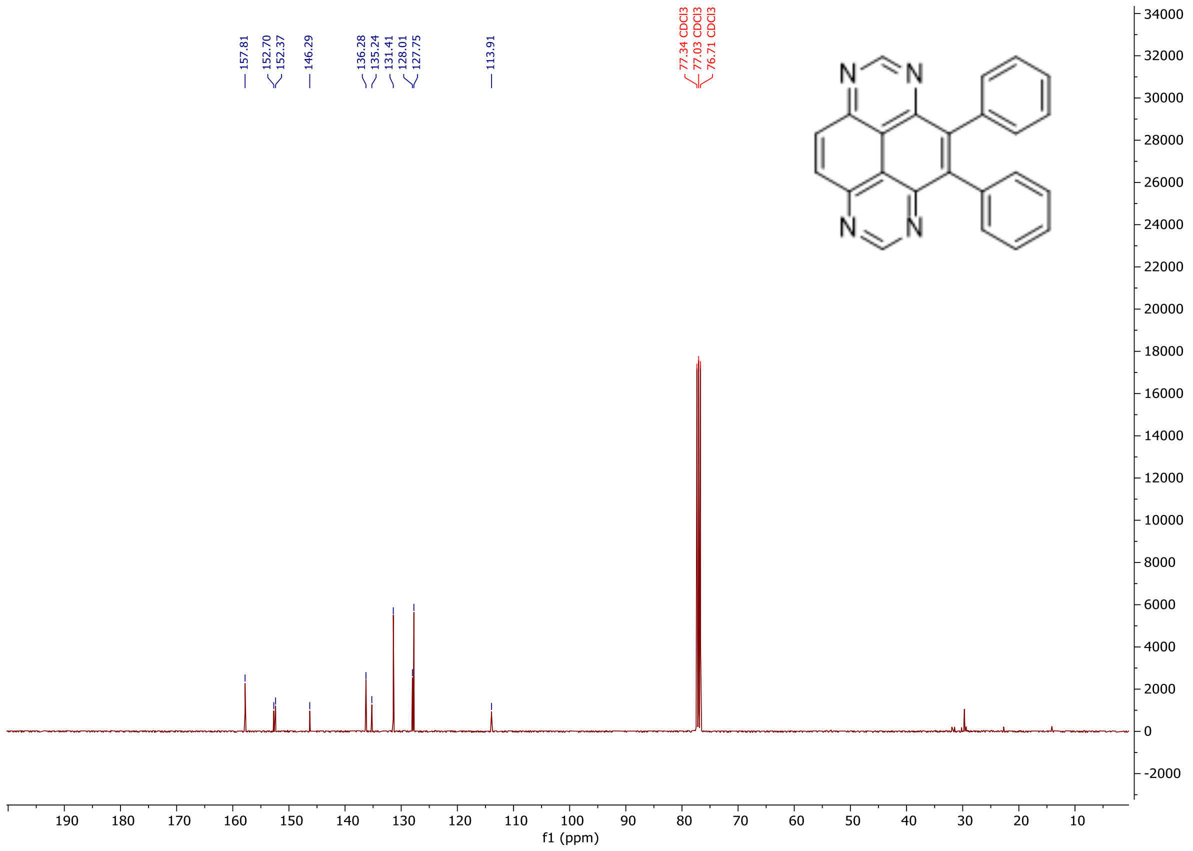

Figure S18: ^{13}C NMR spectrum of **2P-TAP-2**.

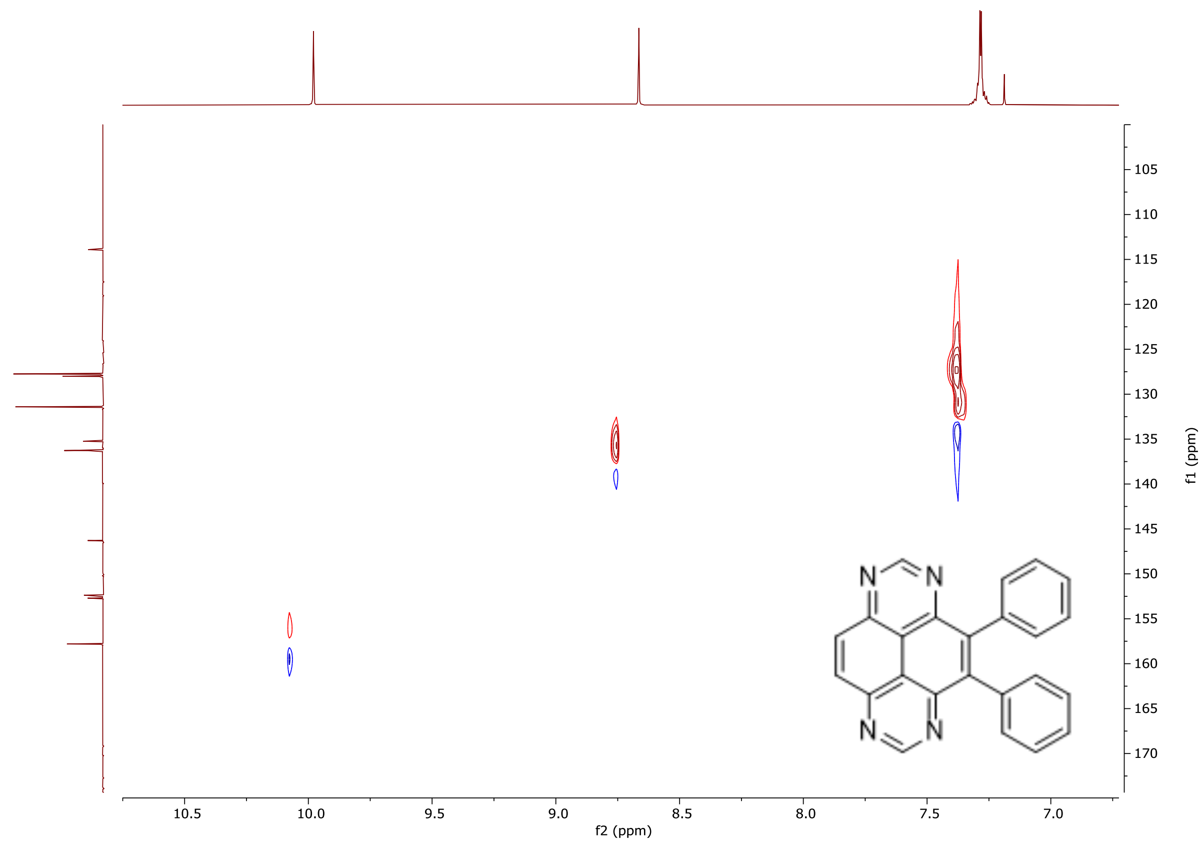

Figure S19: ^{13}C/^{1}H-HSQC NMR spectrum of **2P-TAP-2**.

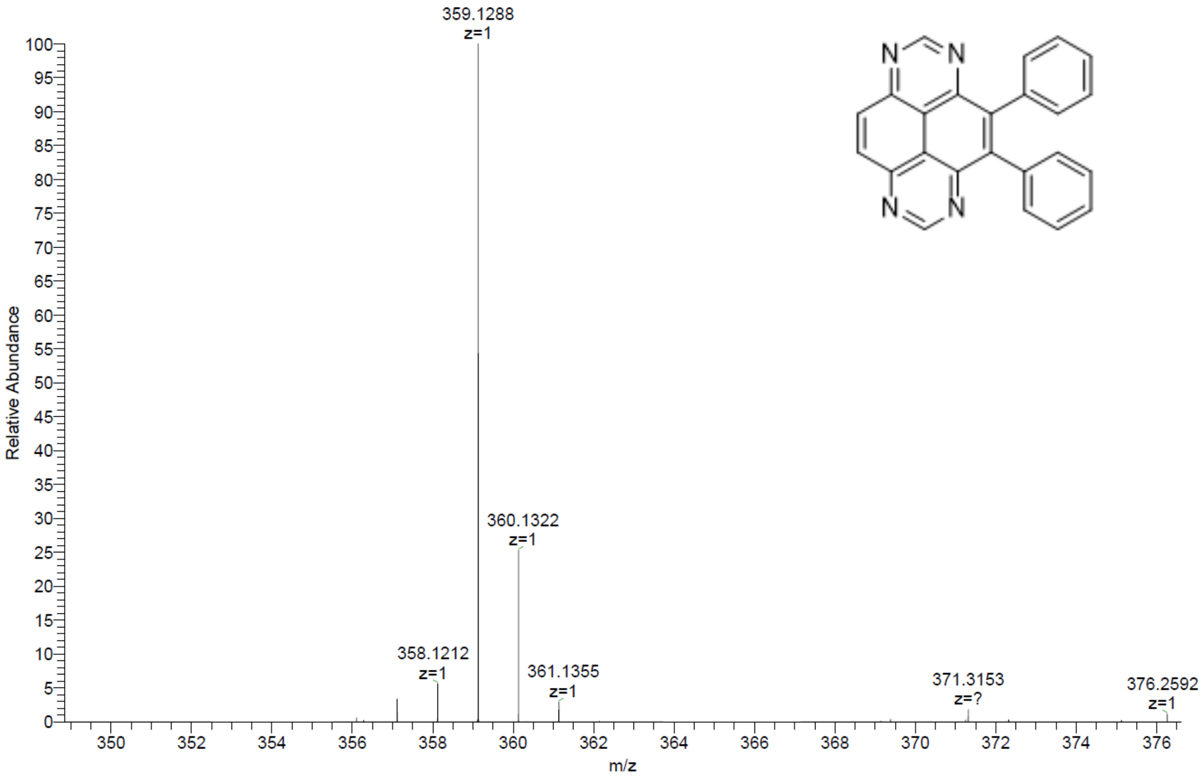

Figure S20: HR-MS (ESI, positive) spectrum of **2P-TAP-2**.

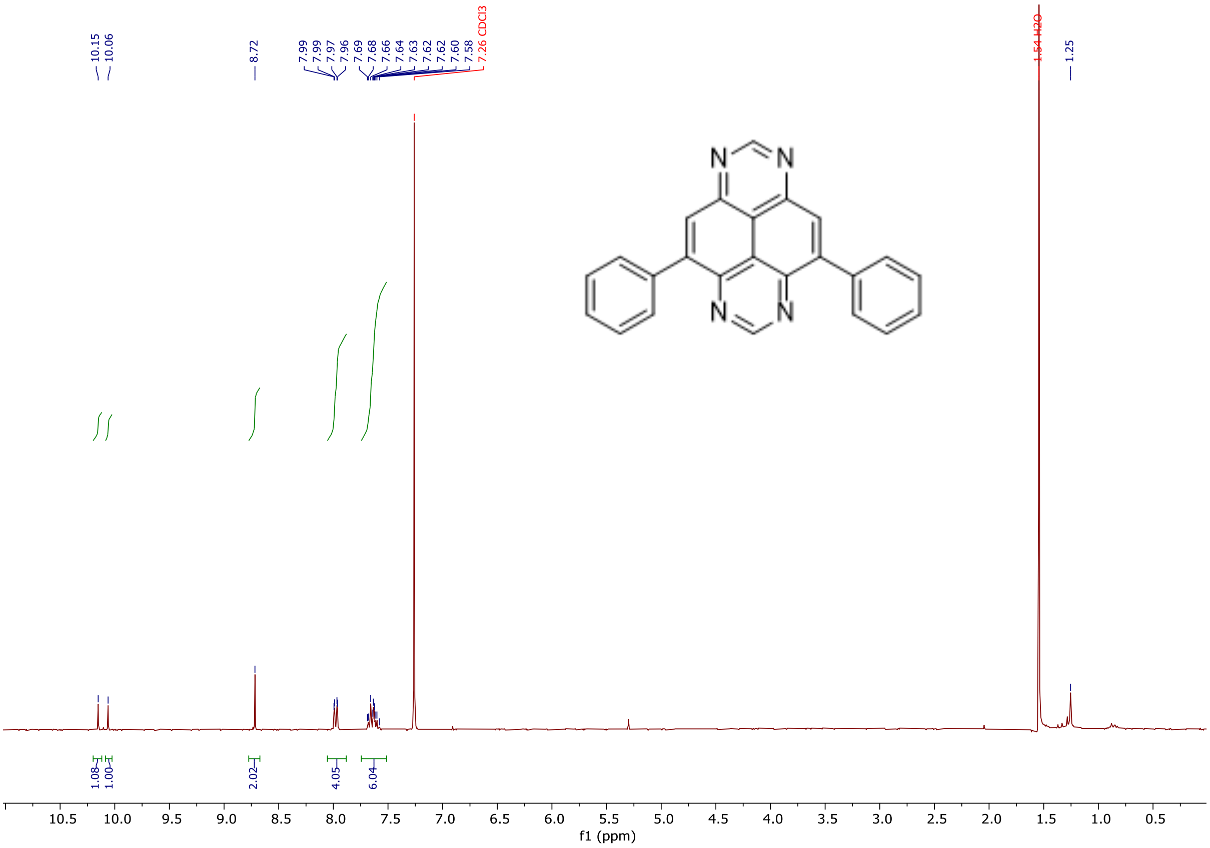

Figure S21: ^{1}H NMR spectrum of **2P-TAP-3**.

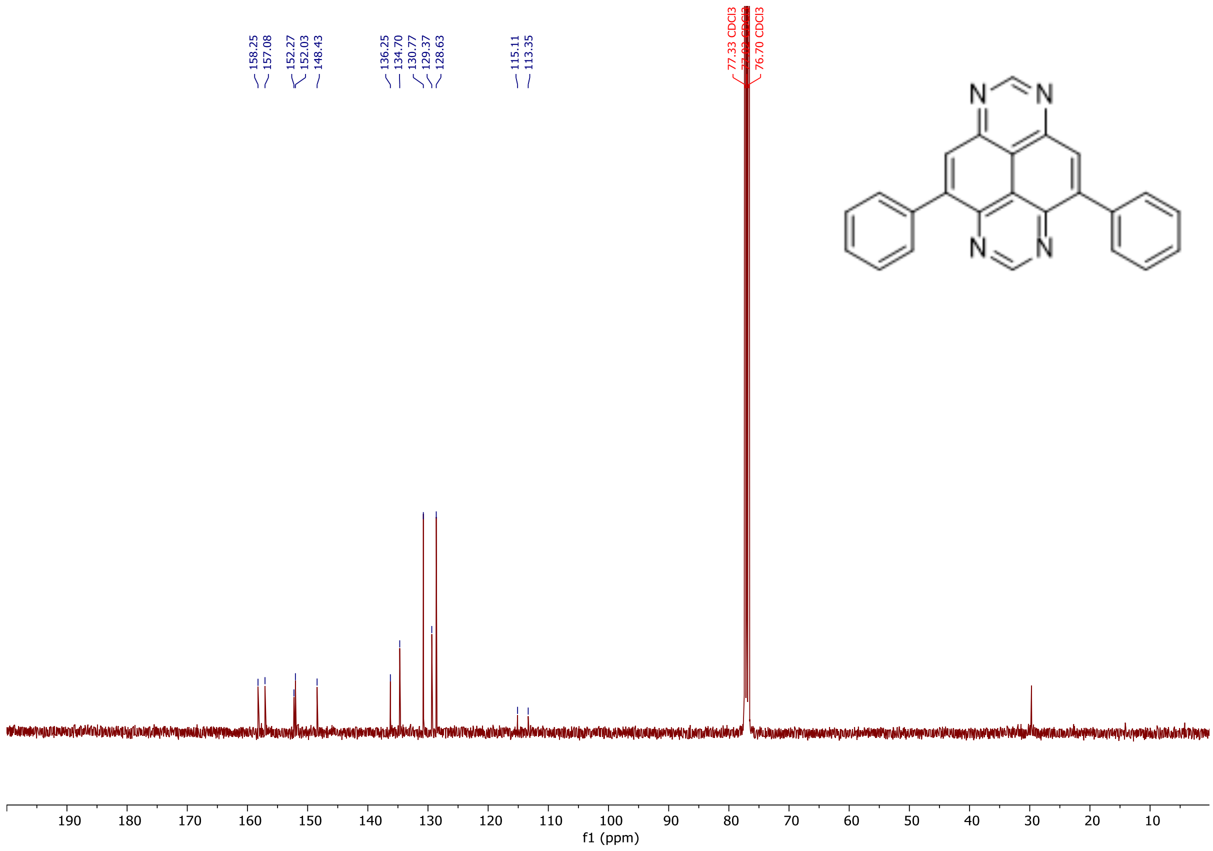

Figure S22: ^{13}C NMR spectrum of **2P-TAP-3**.

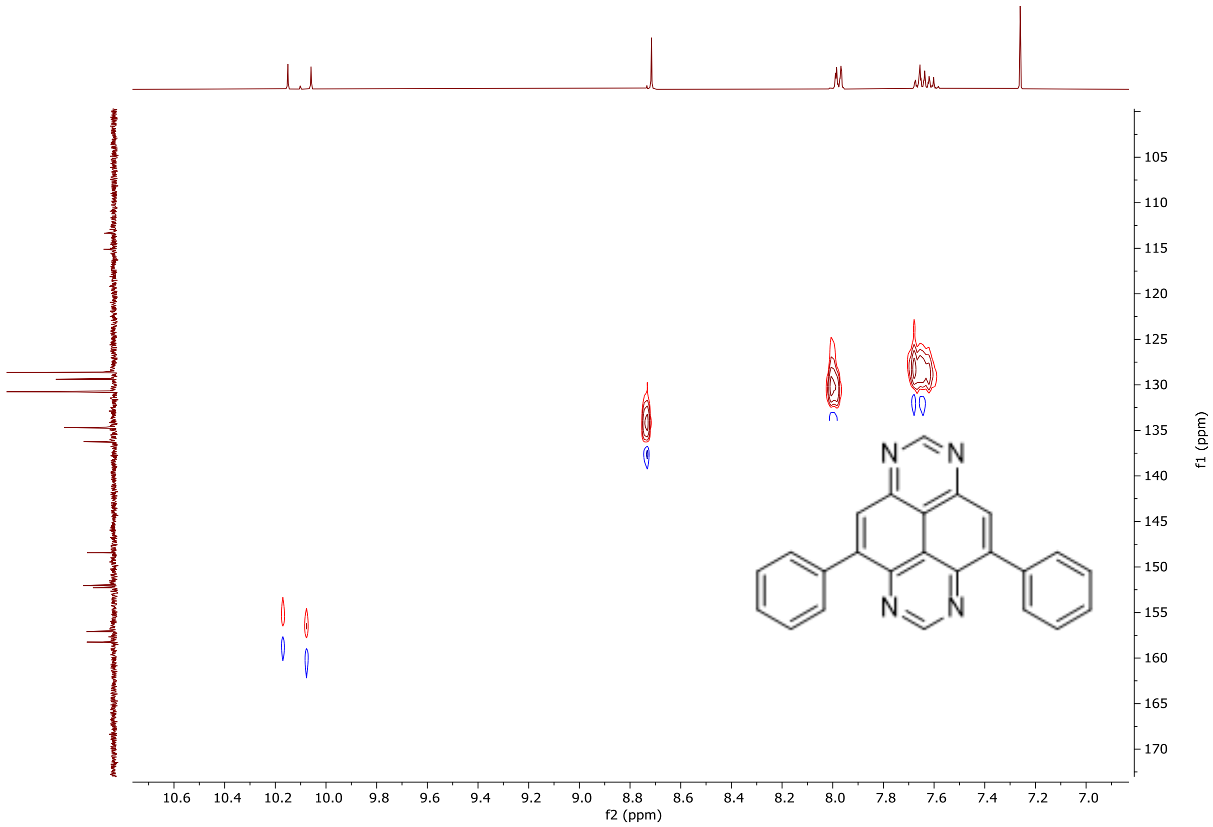

Figure S23: ^{13}C/^{1}H-HSQC NMR spectrum of **2P-TAP-3**.

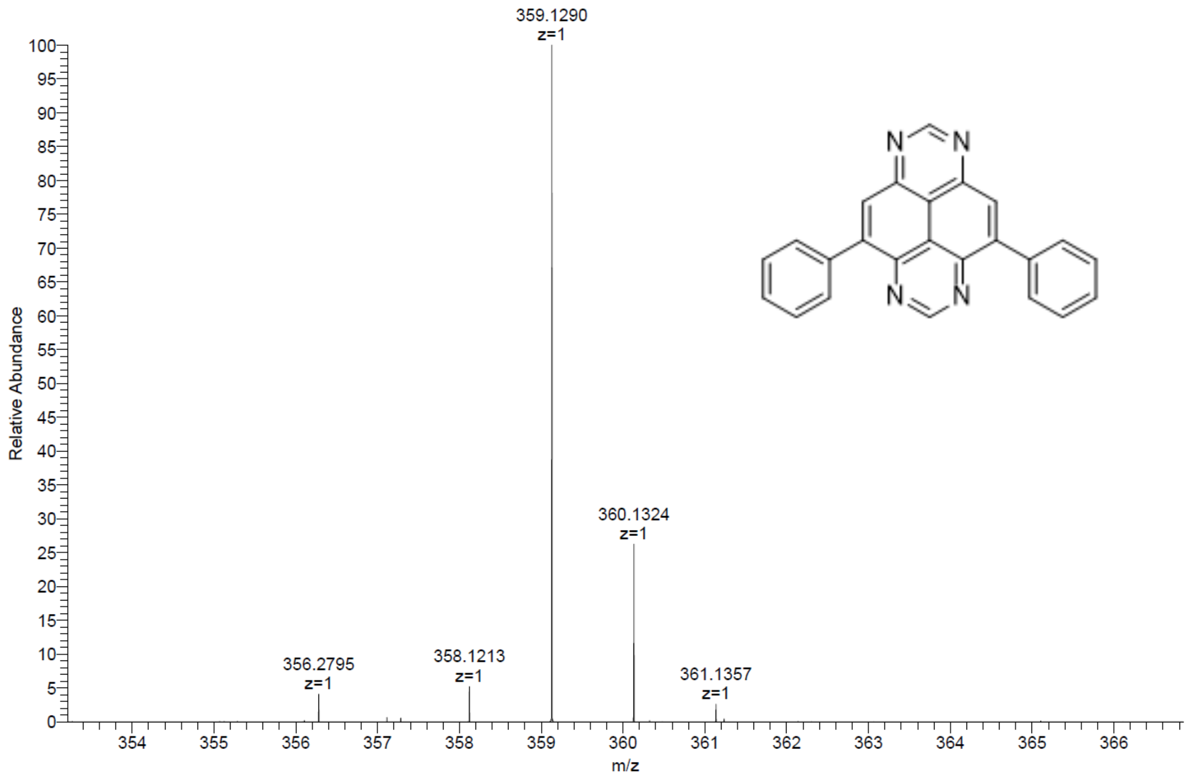

Figure S24: HR-MS (ESI, positive) spectrum of **2P-TAP-3**.

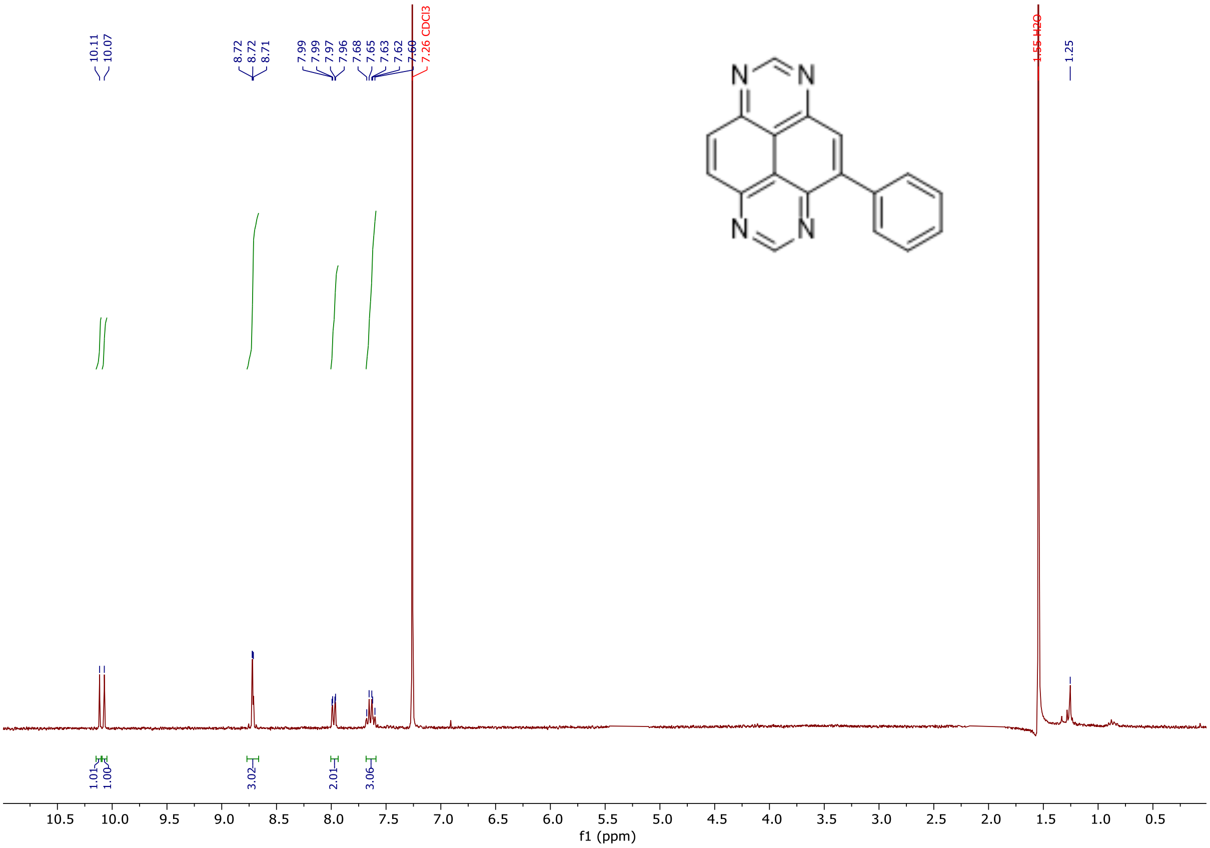

Figure S25: ^{1}H NMR spectrum of **1P-TAP**.

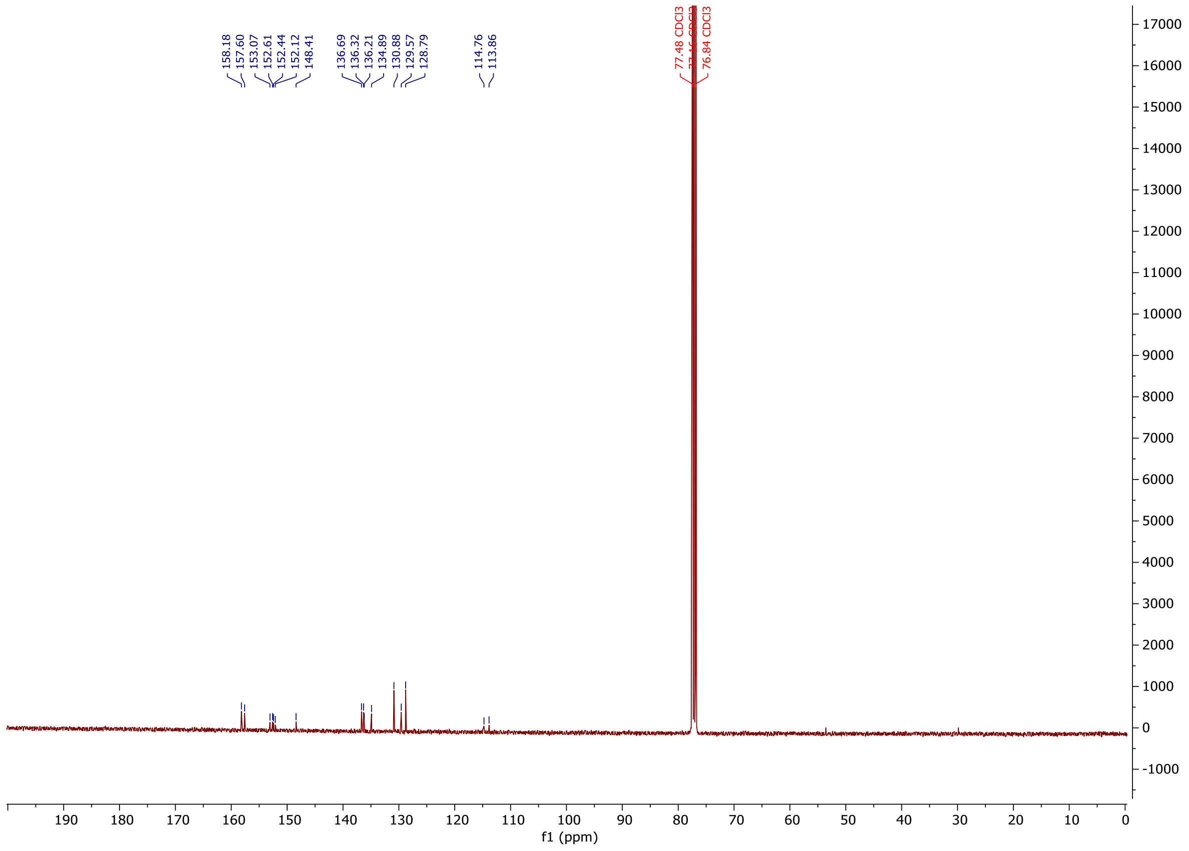

Figure S26: ^{13}C NMR spectrum of **1P-TAP**.

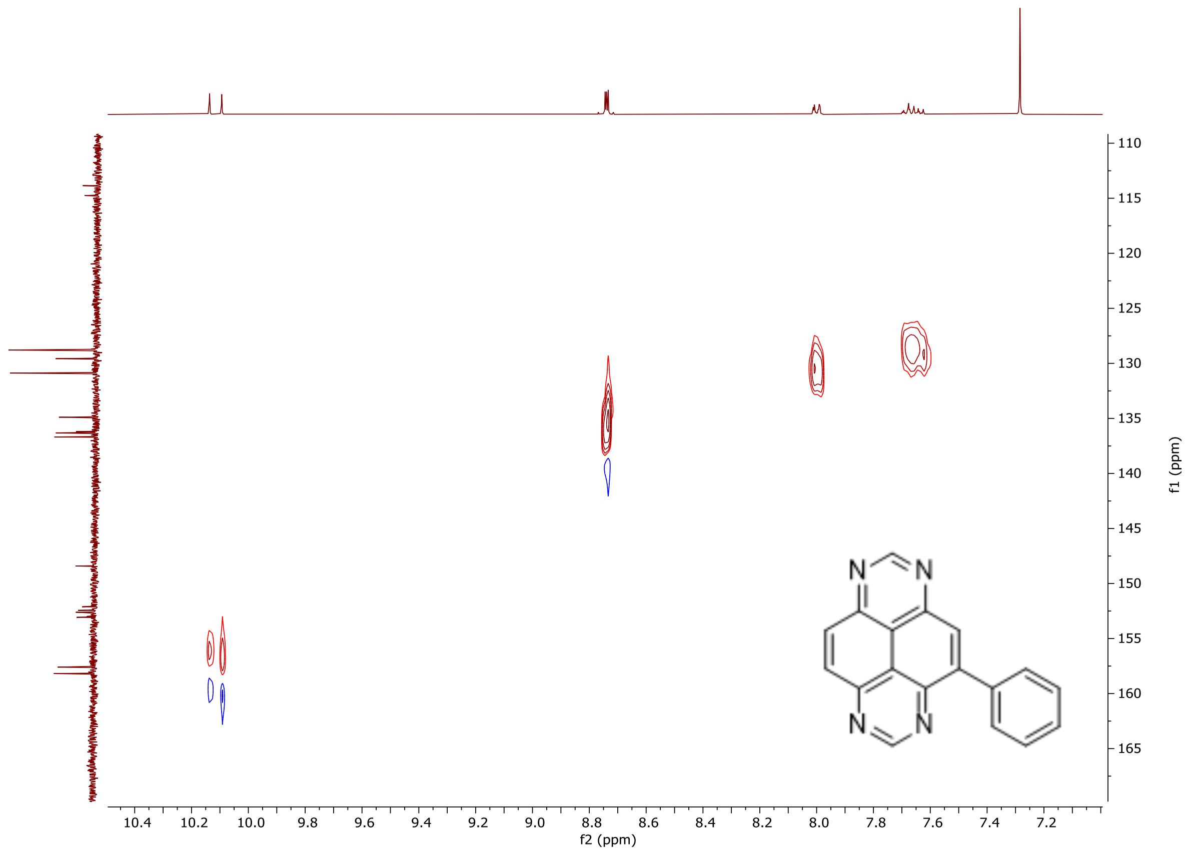

Figure S27: ^{13}C/^{1}H-HSQC NMR spectrum of **1P-TAP**.

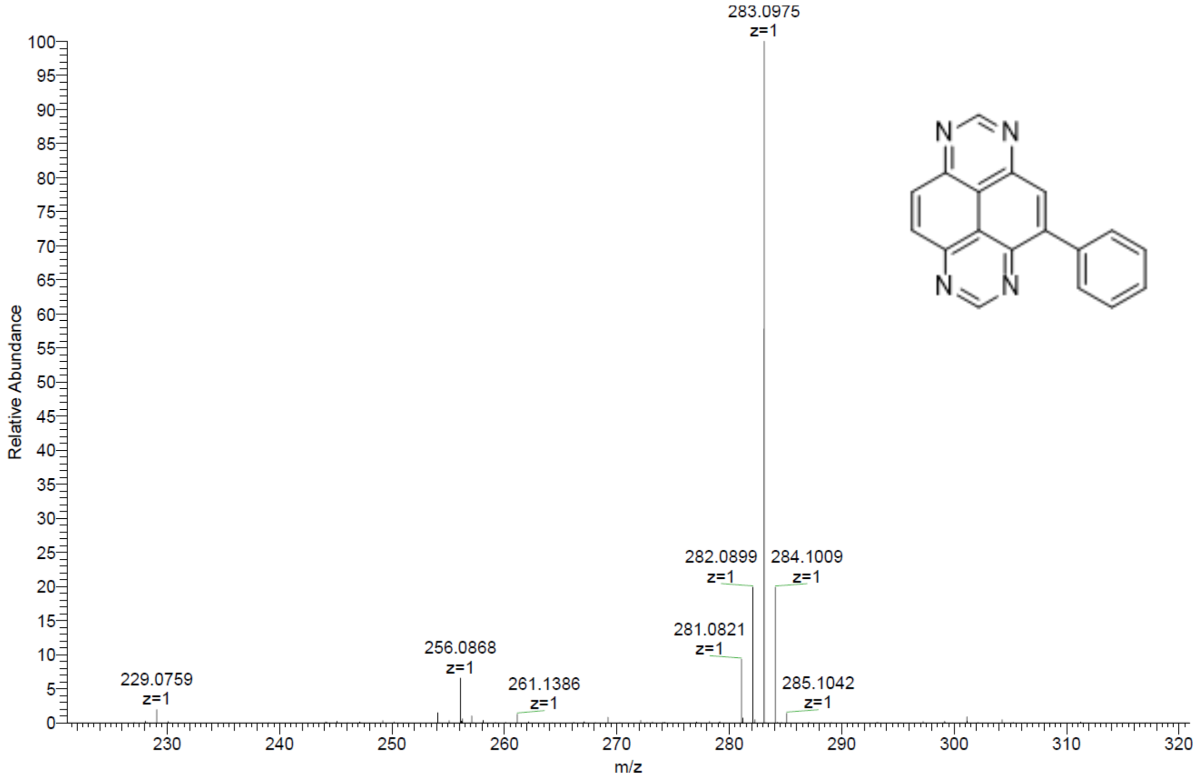

Figure S28: HR-MS (ESI, positive) spectrum of **1P-TAP**.
